# Machine Learning to Prioritize High-Severity Patient Safety Events for Institutional Investigation: Algorithm Development and Validation Study

**DOI:** 10.2196/101393

**Published:** 2026-09-29

**Authors:** Hongbo Chen, Shehnaz Islam, Laura D Pozzobon, Lucas B Chartier, Eldan Cohen

**Affiliations:** 1 Department of Mechanical & Industrial Engineering University of Toronto Toronto, ON Canada; 2 Cerebras Systems Toronto, ON Canada; 3 Health Quality Programs Queen's University Kingston, ON Canada; 4 Quality & Safety Department University Health Network Toronto, ON Canada; 5 Lawerence Bloomberg School of Nursing University of Toronto Toronto, ON Canada; 6 Division of Population Medicine Cardiff University Cardiff, Wales United Kingdom; 7 Department of Medicine University of Toronto Toronto, ON Canada

**Keywords:** AI in health care, clinical decision support, incident reporting systems, learning-to-rank, machine learning, natural language processing, patient safety events, severity triage

## Abstract

**Background:**

Patient safety events (PSEs) are preventable incidents that cause, or have the potential to cause, harm to patients during their medical journey. Although incident reporting systems capture large volumes of such events, only a small proportion undergo comprehensive investigation due to the resource-intensive nature of the review process. Patient safety specialists are tasked with triaging PSEs to prioritize high-severity cases that warrant timely institutional investigation; however, the rapidly increasing volume of events has made manual triage increasingly impractical.

**Objective:**

This study aimed to develop and validate ranking-based machine learning models for prioritizing high-severity PSEs and to compare their performance with conventional classification-based models and the reporter-assigned severity baseline.

**Methods:**

A total of 101,239 PSE reports were retrospectively extracted from a large Canadian academic health system between January 2017 and March 2025. Each report included a free-text incident description and a corresponding severity label denoting the level of severity, assigned through an institutional review process. Both feature-based and transformer-based models were developed using ranking and classification frameworks. Model performance was evaluated using ranking metrics, including average precision and normalized discounted cumulative gain (NDCG) at multiple cutoff thresholds (k=10, 20, 50, 100, 200), as well as Precision@k and Recall@k. The best-performing model was further benchmarked against the reporter-assigned severity baseline.

**Results:**

The ranking model, LLAMA3.1-RA, achieved the highest mean average precision of 0.94 (95% CI 0.90-0.98), representing a 22.1% relative improvement over the best classification model (LLAMA3.1-CL: mean 0.77, 95% CI 0.72-0.82; *P*<.001). LLAMA3.1-RA demonstrated superior performance across 10 of 16 evaluation metrics, with larger relative gains at broader evaluation cutoffs (eg, +12.9% in NDCG@200, +21.1% in Precision@200, and +20.3% in Recall@200). Compared with the reporter-assigned severity baseline, LLAMA3.1-RA achieved significantly higher performance across all 16 metrics, retrieving 83.0% of high-severity PSEs within the top 200 ranked events, compared with 41.0% retrieved under the reporter-assigned severity baseline.

**Conclusions:**

LLAMA3.1-RA demonstrated superior capability in prioritizing high-severity PSEs compared with both classification approaches and the reporter-assigned severity baseline. Integrating this model into triage workflows may offer a scalable and efficient solution for identifying and prioritizing high-severity safety events.

## Introduction

### Overview

Systematic reporting and analysis of adverse events are essential for improving patient safety in modern health care systems. According to the World Health Organization, adverse events are incidents associated with health care that result in, or have the potential to result in, harm to a patient, many of which are potentially preventable [1,2]. Common examples include medication errors, unsafe surgical procedures, and diagnostic errors [3,4]. In Canada, recent national data indicate that unintended harm occurs in approximately 6% of hospitalizations, corresponding to about 1 in every 17 hospital stays, and that multiple harmful events occur in roughly one-quarter of these cases [5]. Beyond clinical consequences, adverse events also impose substantial economic burdens. The indirect costs of unsafe care were estimated at US $118 trillion globally between 2000 and 2020 [6].

The significant impact of adverse events has led many countries to implement mandatory reporting legislation [7,8]. As of 2021, 9 out of 13 jurisdictions in Canada require health care providers to report adverse events [9]. Systematic reporting enables institutions to detect unsafe practices and implement preventive strategies. Adverse events are typically documented as patient safety event (PSE) reports through institutional incident reporting systems (IRSs) [10,11]. PSE reports typically comprise both unstructured fields—such as narrative descriptions of the incident—and structured fields, including the date, location, event type, and severity of harm [12,13]. The submitted reports are investigated by designated hospital personnel, such as risk managers, patient safety specialists, nurse managers, and physicians, to identify contributing factors and root causes [14-16]. Although these reports offer valuable learning opportunities, their investigation is resource-intensive, demanding substantial time, expert knowledge, and interdepartmental coordination [17]. As a result, only a limited proportion of PSE reports undergo comprehensive investigations [18].

Hospitals prioritize the investigation of high-severity PSEs to optimize the effectiveness of limited resources [18,19]. The prioritization of high-severity PSEs is also driven by legislated investigation requirements, as well as ethical obligations to address patient harm, support affected individuals and families, and prevent recurrence through system-level mitigation [14,20]. Event reporters typically assign a severity rating within the IRS to reflect the perceived seriousness of an event. These ratings inform prioritization decisions for investigation efforts. However, research has shown that severity ratings vary substantially among health care professionals, due to differences in clinical roles and familiarity with the severity taxonomy [21,22]. To address this inconsistency, patient safety specialists frequently conduct retrospective manual reviews of submitted reports to identify high-severity cases that warrant timely investigation [18,23,24]. Although individual reports can be assessed through expert review, the rapidly growing volume of submissions makes the timely screening of every incoming report increasingly resource-intensive. For example, over 4.4 million PSE reports were submitted to the National Reporting and Learning System in England between 2020 and 2022 [25]. This operational challenge underscores the need for scalable triage solutions that can support report prioritization while allowing clinical and patient safety resources to be directed toward investigation and quality improvement initiatives.

Natural language processing (NLP) may help address this need by enabling incident descriptions to be analyzed automatically at scale. Accordingly, recent studies have applied NLP techniques to classify the severity of PSE reports [1,18,26,27]. Ong et al [18] used naïve Bayes and support vector machine (SVM) models for severity classification, while Wang et al [27] investigated convolutional neural networks for predicting severity levels. However, most prior studies have relied on classification-based approaches that focus on maximizing categorical accuracy, which may not fully align with the practical needs of patient safety specialists. In institutional settings, these specialists are responsible for prioritizing high-severity PSEs for investigation under significant time constraints and limited resources. In this context, accurately ranking the most critical events at the top of the review list can more effectively support their workflow and decision-making processes.

Ranking-based approaches explicitly learn the relative ordering of instances according to their importance or relevance, making them particularly suitable for this prioritization task. Instead of assigning each report to a discrete severity level, these methods estimate a continuous severity score and order events from most to least severe. By emphasizing relative priority rather than categorical accuracy, ranking-based models align more closely with the operational needs of patient safety specialists. To our knowledge, no prior studies have systematically evaluated ranking approaches for prioritizing PSEs by severity.

### Study Objectives

This study aimed to evaluate the effectiveness of machine learning models for prioritizing high-severity PSEs using retrospectively collected incident reports. We developed both ranking-based and classification-based models and compared their performance in prioritizing PSEs by severity. The top-performing model was further evaluated against the reporter-assigned severity baseline to assess its potential to improve severity triage practices.

## Methods

### Ethical Considerations

This study conducted a secondary analysis of deidentified PSE data collected through institutional IRSs for quality and safety improvement. The study protocol was approved by the hospital’s research ethics board (REB# 23-5237) and was further reviewed to ensure compliance with jurisdictional privacy legislation and institutional quality assurance policies. Following extraction, all data were fully anonymized in accordance with applicable privacy regulations. Informed consent was not required, as the study involved secondary use of anonymized data. No compensation was applicable for the use of these data. Figures and supplementary materials in this manuscript do not contain any identifiable information regarding individual participants or users.

### Study Design

This study aimed to develop and validate machine learning models for prioritizing high-severity PSEs. Figure 1 presents an overview of the study workflow, comprising two phases: (1) data extraction and partitioning and (2) model development and validation.

**Figure 1:**
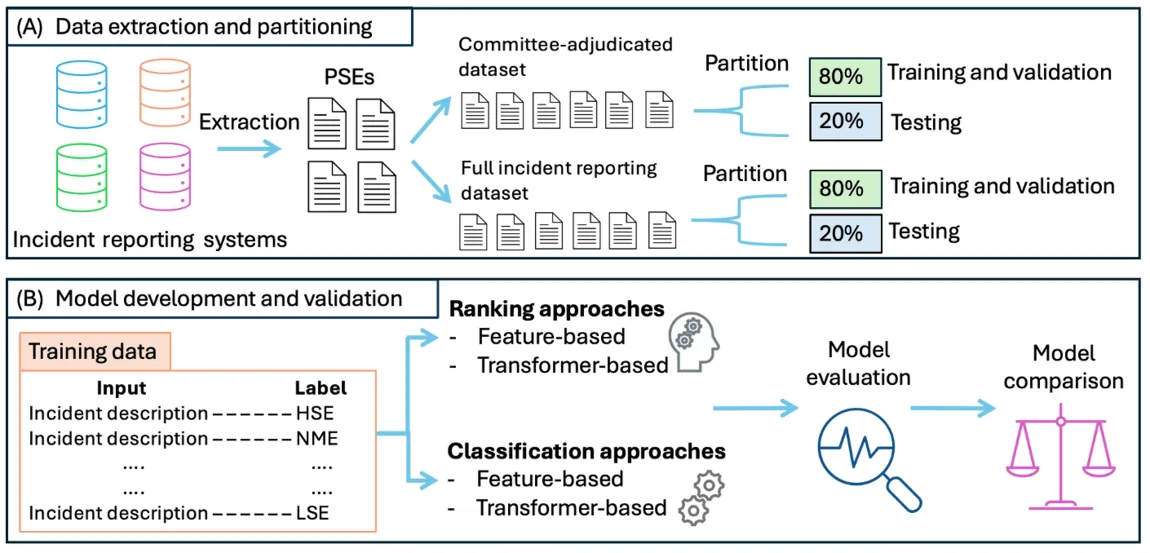
Overview of the study workflow, including data processing, model development, and validation. HSE: high-severity event; LSE: low-severity event; NME: near-miss event.

### Dataset Description

This study used deidentified PSE reports collected from a large academic health system in Eastern Canada. A total of 101,239 reports were extracted from institutional IRSs between January 2017 and March 2025. Each PSE was assigned a severity label to indicate the level of harm associated with the event. The classification includes four categories: (1) high-severity event (HSE), (2) low-severity event (LSE), (3) near-miss event (NME), and (4) no safety-concern event (NSE). HSEs refer to incidents associated with substantial patient harm, including life-threatening outcomes or death. LSEs include incidents with limited or no clinically significant harm. NMEs are incidents that had the potential to cause harm but were prevented before reaching the patient. Nonsafety events refer to reports that do not represent a patient safety issue or do not involve a deviation from standard care processes. To further illustrate how event outcomes and clinical circumstances inform severity determination, Multimedia Appendix 1 provides synthetic examples of PSE reports together with the rationale for their corresponding severity classifications.

Severity labeling followed a 2-stage institutional review process. First, patient safety specialists screened PSE reports submitted through the IRS to identify those with potential for high severity. Reports identified through this initial screening were subsequently reviewed by a multidisciplinary committee of point-of-care clinicians, patient safety specialists, operational leaders, and other domain experts. Based on this review, severity labels were assigned according to established institutional classification practices. Although PSE reports describe events that have already occurred, their screening and escalation are ongoing components of the institutional patient safety workflow. Timely screening is important because the late identification of potentially high-severity cases may impede institutional investigation and appropriate follow-up actions. The proposed approaches are intended to support the initial screening stage by prioritizing incoming reports for specialist review and potential escalation. Final severity determination remains the responsibility of the multidisciplinary committee.

We composed 2 analytic datasets to support complementary but distinct evaluation objectives. Table 1 presents the distribution of PSE reports by severity category across the 2 datasets. The first dataset comprised PSE reports that underwent multidisciplinary committee review and received severity labels. These expert-adjudicated labels provide a high-quality reference standard and were used to support robust assessment and comparison of model performance. This dataset included 2675 reports, of which 1037 (38.8%) were classified as HSE (panel A in Table 1). Throughout this study, the term PSE refers to any reported safety-related incident that could have resulted, or did result, in unnecessary harm to a patient, whereas HSE, LSE, NME, and NSE denote the severity categories used in the institutional classification system.

The second dataset included all PSE reports submitted to the IRS, encompassing both committee-reviewed reports with severity labels (ie, the entire committee-adjudicated dataset) and reports that were not escalated for committee review and therefore did not receive severity labels. Under current institutional workflows, reports that are not escalated are implicitly treated as lower-priority events and are not subject to formal severity adjudication. For analytical purposes, we assume that these unreviewed reports represent non-HSEs as operationally defined by existing triage practices. This assumption reflects institutional decision-making rather than asserting the absence of patient safety concerns. It enables operationally relevant evaluation in which the model must prioritize a small number of high-severity PSEs for investigation from a large volume of incoming reports, mirroring real-world safety triage conditions. The second dataset comprised 101,239 reports, of which 1037 (1%) were classified as HSEs, reflecting the low prevalence of high-severity cases in routine triage settings (panel B in Table 1).

**Table 1:** Distribution patient safety event reports by severity labels.

Severity label			Reports, n (%)		
Panel A. Committee-adjudicated dataset					
	HSE^a^	1037 (38.77)			
	LSE^b^	830 (31.03)			
	NME^c^	56 (2.09)			
	NSE^d^	752 (28.11)			
	Total	2675 (100)			
Panel B. Full incident reporting system dataset					
	HSE	1037 (1.00)			
	LSE	830 (0.82)			
	NME	56 (0.06)			
	NSE^e^	99,316 (98.10)			
	Total	101,239 (100)			

^a^HSE: high-severity event.

^b^LSE: low-severity event.

^c^NME: near-miss event.

^d^NSE: no safety-concern event

^e^NSE includes both explicitly classified NSEs and reports not escalated for committee review.

### Model Development and Validation

In this study, we developed machine learning models to prioritize high-severity PSEs using both ranking and classification approaches. We used the incident description section of each PSE report as input and the corresponding severity label as the outcome variable, consistent with prior work [18,26,27]. The incident description is a free-text narrative field in which the event reporter documents the circumstances and outcomes associated with the safety event.

Ranking-based models were developed using a learning-to-rank (LTR) framework, which learns to order items based on their relevance or importance and produces a ranked list [28,29]. During the training of LTR models, the assigned labels guide the model in identifying patterns that distinguish higher-priority items from lower-priority items [28,30]. Once trained, the model generates a continuous priority score for each item, which determines its position in the resulting list. LTR differs from conventional classification in both its objective and output. Classification models aim to assign each item to a predefined category [31], whereas LTR models focus on positioning the highest-priority items near the top of a ranked list. Accordingly, classification models are optimized to predict predefined categories. In this study, the classification models predicted all 4 severity categories, and the predicted probability assigned to the HSE class was used as a continuous score to order reports for ranking-based evaluation. As a sensitivity analysis, we also evaluated a binary classification formulation that directly distinguished HSE from non-HSE reports (Multimedia Appendix 2). In contrast, LTR models are trained directly to optimize the relative ordering of items based on predicted priority. Items within the same category may receive different scores, allowing finer prioritization within categories. This distinction is particularly important when it is impractical to review every available item and attention must be directed toward the highest-priority cases.

In this study, each PSE report represented an item to be ranked, and its institutional severity label indicated its relative priority. Using the incident descriptions, the LTR models learned textual patterns associated with differences in event severity and generated a predicted priority score for each report. The reports were then ordered according to these scores, with high-severity PSEs prioritized for review.

To evaluate the ranking and classification approaches across different model architectures, we implemented both feature-based and transformer-based models. Feature-based models refer to traditional machine learning algorithms that are computationally efficient and frequently serve as baselines in NLP tasks [32,33]. Transformer-based models are deep learning architectures that have achieved state-of-the-art performance in a variety of classification and ranking benchmarks [34-36]. Their effectiveness is attributed to their ability to capture contextual dependencies in text and leverage knowledge from large-scale pretraining.

We trained 2 feature-based models: XGBoost and LightGBM [32,37], both of which are gradient-boosted decision tree algorithms known for their effectiveness in prior ranking applications [29]. To provide comparisons with conventional text classification approaches, we also developed SVM and multinomial naïve Bayes (MNB) classification baselines using bag-of-words (BAG) and term frequency inverse document frequency (TF-IDF) representations. We trained 8 transformer-based models: BERT, ROBERTA, XLM-ROBERTA, BIOBERT, BIOMEDBERT, GATORTRON, LLAMA3.1-8B, and QWEN3-8B [36,38-43]. BERT, ROBERTA, and XLM-ROBERTA were selected due to their widespread adoption and competitive performance across diverse NLP tasks [44-46]. BIOBERT, BIOMEDBERT, and GATORTRON were included for their pretraining on biomedical corpora, optimizing their performance for tasks involving clinical narratives and medical terminology [39-41]. LLAMA3.1-8B was included because it is a widely adopted open-source model that serves as an established benchmark across NLP applications [42]. Qwen3-8B was selected as a more recently developed open-source model representing advances in contemporary large language model architectures [43]. Throughout the manuscript, the suffix “RA” identifies models developed for ranking, whereas “CL” identifies models developed for classification. A complete summary of the evaluated models, abbreviations, and full model names is provided in Multimedia Appendix 3. In addition, for the feature-based models, free-text narratives were converted into structured feature representations before being used as input; these feature-extraction methods are also described in Multimedia Appendix 3.

The model development workflow is illustrated in Figure 2. The dataset was partitioned at the individual-report level, with no report appearing in more than 1 data partition. Patient-level stratification was not performed because the fully deidentified dataset provided under the research ethics board–approved secondary-use protocol did not contain patient identifiers or linkage keys. The dataset was partitioned into 80% for training and validation and 20% for testing, with no overlap between splits. For feature-based models, hyperparameter tuning was conducted using grid search with 5-fold cross-validation [47]. The training and validation data were divided into 5 mutually exclusive folds; for each hyperparameter configuration, models were trained on 4 folds and validated on the fifth, with the process repeated across all 5 folds. The optimal configuration was selected based on the average cross-validated performance, measured by average precision. For transformer-based models, the dataset was partitioned into 70% for training, 10% for validation, and 20% for testing. To ensure comparability, the same testing set used for the feature-based models was retained. Models were trained on the training set, and hyperparameter tuning was conducted on the validation set. A conventional training-validation-test split was used instead of cross-validation due to the substantial computational cost associated with training transformer-based models. A complete list of tuned hyperparameters for both model types is provided in Multimedia Appendix 4.

**Figure 2:**
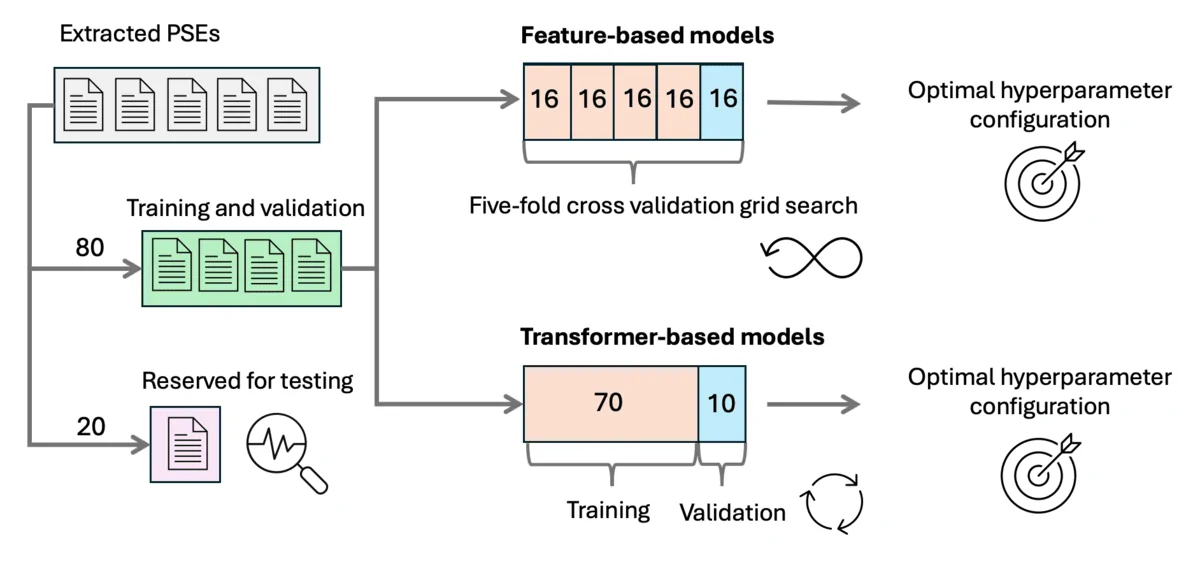
Model development workflow for feature-based and transformer-based models. Numbers indicate the proportion of the dataset allocated to each stage of model training, validation, and testing. PSE: patient safety event.

The distribution of severity labels in the dataset was imbalanced, with some classes underrepresented. The imbalance can negatively impact the performance of classification-based models, particularly for minority classes [48]. For feature-based classifiers, we applied the Synthetic Minority Oversampling Technique (SMOTE) to generate synthetic samples for the underrepresented classes in the training set, thereby addressing class imbalance [49]. SMOTE was applied only to the training set to preserve the original class distribution in the test set and avoid bias in model evaluation. For transformer-based classifiers, we implemented a loss reweighting strategy by assigning higher weights to minority classes in the loss function [48]. This approach penalizes misclassifications of underrepresented classes more heavily, promoting more balanced performance across classes. Within the full IRS dataset, the distribution of severity labels is characterized by extreme class imbalance. To mitigate this imbalance during classifier training, we additionally explored random downsampling of the majority class (ie, NSE). All non-NSE severity reports were retained, while the number of NSEs was varied across a predefined range to construct training sets with differing class imbalance ratios. The downsampling ratio was treated as a hyperparameter and selected based on validation performance. Downsampling was applied exclusively to the training data, and all validation and testing were conducted on datasets reflecting the original class distribution to preserve operationally relevant prevalence. Detailed parameters for SMOTE, random downsampling, and class-weighted loss, together with the numbers of synthetic samples generated and NSE reports removed, and the final model-specific sampling ratios and class weights, are provided in Multimedia Appendix 5. On the other hand, ranking-based models focus on learning the relative ordering of items rather than predicting discrete labels. These models are optimized based on comparisons between items, making them inherently less sensitive to class imbalance [50].

### Model Evaluation and Comparison

Model performance was evaluated using established ranking metrics, including average precision, normalized discounted cumulative gain (NDCG) at multiple cutoff thresholds (k=10, 20, 50, 100, 200), as well as precision and recall computed at the same cutoffs. Average precision quantifies the model’s ability to rank high-severity PSEs near the top of the list and is calculated as the mean of precision values at each position where a high-severity PSE is retrieved. NDCG measures the quality of a ranked list by accounting for both the severity of the PSEs and their rank positions, offering a comprehensive evaluation of ranking effectiveness [29]. Precision@k denotes the proportion of high-severity PSEs ranked in the top k positions, while recall@k captures the proportion of all high-severity PSEs that appear within the top k. High-severity PSEs were defined as those assigned a severity label of HSE, which necessitates prioritization for institutional investigation. All evaluation metrics range from 0 to 1, with higher values indicating better ranking performance. The mathematical definitions of all evaluation metrics are provided in Multimedia Appendix 6.

During testing, the models were evaluated based on their ability to rank PSE reports according to severity. Specifically, each model generated an ordered list in which reports with higher predicted severity were placed closer to the top, and those with lower predicted severity were placed further down the list. The original HSE, LSE, NME, and NSE labels were retained throughout model testing. We used 2 complementary sets of evaluation metrics. Average precision, Precision@k, and Recall@k used binary relevance to evaluate the study’s primary objective of prioritizing HSE reports for institutional investigation. For these metrics only, HSE reports were considered relevant, whereas LSE, NME, and NSE reports were considered nonrelevant. In contrast, NDCG used graded relevance, with HSE, LSE, NME, and NSE assigned decreasing levels of priority, to evaluate the relative ordering of reports across the complete severity spectrum. Together, these metrics evaluated both HSE prioritization and the preservation of the broader ordering of event severity.

For classification models, PSEs were ranked by their predicted probabilities of receiving a severity label of HSE and evaluated performance using ranking metrics. This approach more effectively captures the model’s ability to prioritize high-severity PSEs than conventional classification metrics. For example, patient safety teams may triage the top 50-100 PSEs that are relatively more severe than others. In such contexts, ranking-based evaluation directly assesses the model’s ability to order events by clinical urgency, thereby aligning more closely with clinical workflows.

We evaluated all trained models on the internal testing set, which comprised 20% of the total PSEs. The 95% CI was computed using bootstrapping with 2000 random subsamples, drawn with replacement and matched to the testing set’s original size, to quantify uncertainty in model performance. We compared the performance of the best-performing ranking and classification models using paired *t* tests. In addition, the top-performing model was compared with the reporter-assigned severity baseline. This baseline was derived from the preliminary severity ratings selected by frontline health care workers when submitting reports to the IRS. These ratings reflect reporters’ initial perceptions and are distinct from the gold-standard severity labels subsequently assigned through multidisciplinary committee adjudication. Baseline performance was evaluated by ranking reports according to reporter-assigned severity and comparing the resulting rankings against the reference severity labels using the same ranking metrics applied to the developed models. To obtain a more robust estimate of baseline performance, the baseline rankings were bootstrapped using the same resampling procedure as the model evaluations. Multiple-comparison adjustment was performed using a Bonferroni correction, setting the significance threshold at *P*<.001 (0.05/64) to maintain a family-wise error rate of 0.05 across 64 tests (16 metrics × 4 comparisons).

## Results

### Performance Comparison of Classification and Ranking Models

Among all ranking models, LLAMA3.1-RA achieved the highest performance based on average precision, while LLAMA3.1-CL demonstrated the best performance among the classification models. Table 2 summarizes the comparative results between these 2 top-performing models, while Figure 3 visualizes their NDCG@k, Precision@k, and Recall@k across the evaluated cutoff values. Average precision was selected as the primary criterion for model selection, as it provides a comprehensive measure of each model’s overall ranking capability by jointly accounting for the accuracy and ordering of high-severity PSEs across the ranked list. Detailed performance metrics for all developed models are presented in Multimedia Appendix 7. To provide additional benchmarks against conventional text-classification approaches, SVM and MNB classifiers using BAG and TF-IDF representations were also evaluated, with detailed results presented in Multimedia Appendix 8. Among these traditional baselines, SVM-BAG achieved the highest average precision in the committee-adjudicated dataset (mean 0.62, 95% CI 0.55-0.69), while MNB–TF-IDF achieved the highest average precision in the full IRS dataset (mean 0.49, 95% CI 0.44-0.54). In both evaluation settings, the best-performing traditional baseline achieved lower average precision than LLAMA3.1-CL and LLAMA3.1-RA.

As shown in Table 2 and Figure 3, LLAMA3.1-RA achieved equal or higher mean performance than LLAMA3.1-CL across all 16 evaluation metrics in panel A (committee-adjudicated dataset). LLAMA3.1-RA demonstrated a significantly higher average precision (mean 0.94, 95% CI 0.90-0.98) than LLAMA3.1-CL (mean 0.77, 95% CI 0.72-0.82; *P*<.001), corresponding to a 22.1% relative improvement. Both models achieved perfect performance at the most restrictive cutoffs (NDCG@10-20=1.00; Precision@10-20=1.00), with identical Recall@10 (0.05) and Recall@20 (0.10), indicating comparable ability to identify the most severe events within the top-ranked positions. Performance differences became more pronounced at broader cutoffs. At k=100, LLAMA3.1-RA improved NDCG by 4.3%, precision by 9.1%, and recall by 9.3%. At k=200, relative improvements increased to 12.9% for NDCG, 21.1% for precision, and 20.3% for recall (all *P*<.001). Overall, LLAMA3.1-RA demonstrated statistically significant advantages in 10 of the 16 evaluated metrics, with the largest gains observed at higher cutoffs, reflecting more consistent prioritization of high-severity PSEs across the ranked list. In Panel B (full IRS dataset), the superiority of the ranking model remained consistent despite the substantially lower prevalence of high-severity PSEs. LLAMA3.1-RA achieved a 27.1% relative improvement in average precision (mean 0.89, 95% CI 0.84-0.94 vs mean 0.70, 95% CI 0.64-0.76; *P*<.001). Similar to panel A, substantial gains were observed at broader cutoffs, particularly at k=200, where relative improvements reached 15.9% for NDCG, 24.6% for precision, and 22.2% for recall (all *P*<.001). An aggregate error and model-disagreement analysis was also conducted at the top-200 cutoff to examine HSE reports retrieved by both models, by either model alone, or by neither model, together with the severity-category distribution of the non-HSE reports included within each model’s top 200 positions; complete results are provided in Multimedia Appendix 9. Additional ablation analyses examining the effects of training-set size and learning objective on LLAMA3.1 performance are presented in Multimedia Appendix 10. To assess temporal robustness, we additionally evaluated LLAMA3.1-RA using chronological data partitioning, with the earliest 70% of reports used for training, the subsequent 10% for validation, and the most recent 20% for testing. The model maintained strong performance under temporal validation, achieving an average precision of 0.92 and NDCG@200, Precision@200, and Recall@200 values of 0.94, 0.84, and 0.81, respectively. These results indicate that LLAMA3.1-RA maintained broadly stable prioritization performance when trained on earlier reports and evaluated on more recent reports. Complete methods and results are provided in Multimedia Appendix 11.

**Table 2:** Comparative performance of the best-performing classification model (LLAMA3.1-CL) and ranking model (LLAMA3.1-RA) across 2 evaluation settings: (panel A) the committee-adjudicated dataset, and (panel B) the full incident reporting system dataset reflecting real-world operationally representative conditions. Performance values are reported as mean (95% CI) on the held-out testing set, estimated using 2000 bootstrap resamples. Relative improvement (%) represents the proportional performance gain of the ranking model over the classification model. *P* values indicate statistical significance based on paired comparisons with Bonferroni correction.

Metrics			LLAMA3.1-CL, mean (95% CI)		LLAMA3.1-RA, mean (95% CI)		Relative improvement (%)		*P* value
Panel A. Committee-adjudicated dataset									
	Average precision	0.77 (0.72-0.82)		0.94 (0.90-0.98)		22.1		<.001	
	NDCG@10	1.00 (0.98-1.00)		1.00 (1.00-1.00)		0.0		>.99	
	NDCG@20	1.00 (0.96-1.00)		1.00 (0.99-1.00)		0.0		>.99	
	NDCG@50	0.96 (0.91-1.00)		0.99 (0.97-1.00)		3.1		<.001	
	NDCG@100	0.94 (0.91-0.97)		0.98 (0.94-1.00)		4.3		<.001	
	NDCG@200	0.85 (0.80-0.90)		0.96 (0.93-0.99)		12.9		<.001	
	Precision@10	1.00 (0.95-1.00)		1.00 (1.00-1.00)		0.0		>.99	
	Precision@20	1.00 (0.94-1.00)		1.00 (0.98-1.00)		0.0		>.99	
	Precision@50	0.92 (0.83-1.00)		0.98 (0.92-1.00)		6.5		<.001	
	Precision@100	0.88 (0.82-0.94)		0.96 (0.91-1.00)		9.1		<.001	
	Precision@200	0.71 (0.66-0.76)		0.86 (0.80-0.92)		21.1		<.001	
	Recall@10	0.05 (0.04-0.06)		0.05 (0.04-0.06)		0.0		>.99	
	Recall@20	0.10 (0.09-0.11)		0.10 (0.09-0.11)		0.0		>.99	
	Recall@50	0.22 (0.19-0.25)		0.24 (0.21-0.27)		9.1		<.001	
	Recall@100	0.43 (0.39-0.47)		0.47 (0.43-0.51)		9.3		<.001	
	Recall@200	0.69 (0.64-0.74)		0.83 (0.79-0.87)		20.3		<.001	
Panel B. Full incident reporting system dataset									
	Average precision	0.70 (0.64-0.76)		0.89 (0.84-0.94)		27.1		<.001	
	NDCG@10	0.96 (0.82-1.00)		1.00 (1.00-1.00)		4.2		<.001	
	NDCG@20	0.94 (0.79-1.00)		1.00 (0.99-1.00)		6.4		<.001	
	NDCG@50	0.92 (0.85-0.99)		0.99 (0.98-1.00)		7.6		<.001	
	NDCG@100	0.90 (0.85-0.95)		0.98 (0.96-1.00)		8.9		<.001	
	NDCG@200	0.82 (0.78-0.86)		0.95 (0.92-0.98)		15.9		<.001	
	Precision@10	0.90 (0.74-1.00)		1.00 (1.00-1.00)		11.1		<.001	
	Precision@20	0.90 (0.76-1.00)		1.00 (0.98-1.00)		11.1		<.001	
	Precision@50	0.88 (0.81-0.95)		0.98 (0.94-1.00)		11.4		<.001	
	Precision@100	0.86 (0.80-0.92)		0.95 (0.90-1.00)		10.5		<.001	
	Precision@200	0.65 (0.59-0.71)		0.81 (0.74-0.88)		24.6		<.001	
	Recall@10	0.04 (0.03-0.05)		0.05 (0.04-0.06)		25.0		<.001	
	Recall@20	0.09 (0.07-0.11)		0.10 (0.09-0.11)		11.1		<.001	
	Recall@50	0.22 (0.19-0.25)		0.24 (0.21-0.27)		9.1		<.001	
	Recall@100	0.42 (0.38-0.46)		0.46 (0.41-0.51)		9.5		<.001	
	Recall@200	0.63 (0.58-0.68)		0.77 (0.72-0.82)		22.2		<.001	

**Figure 3:**
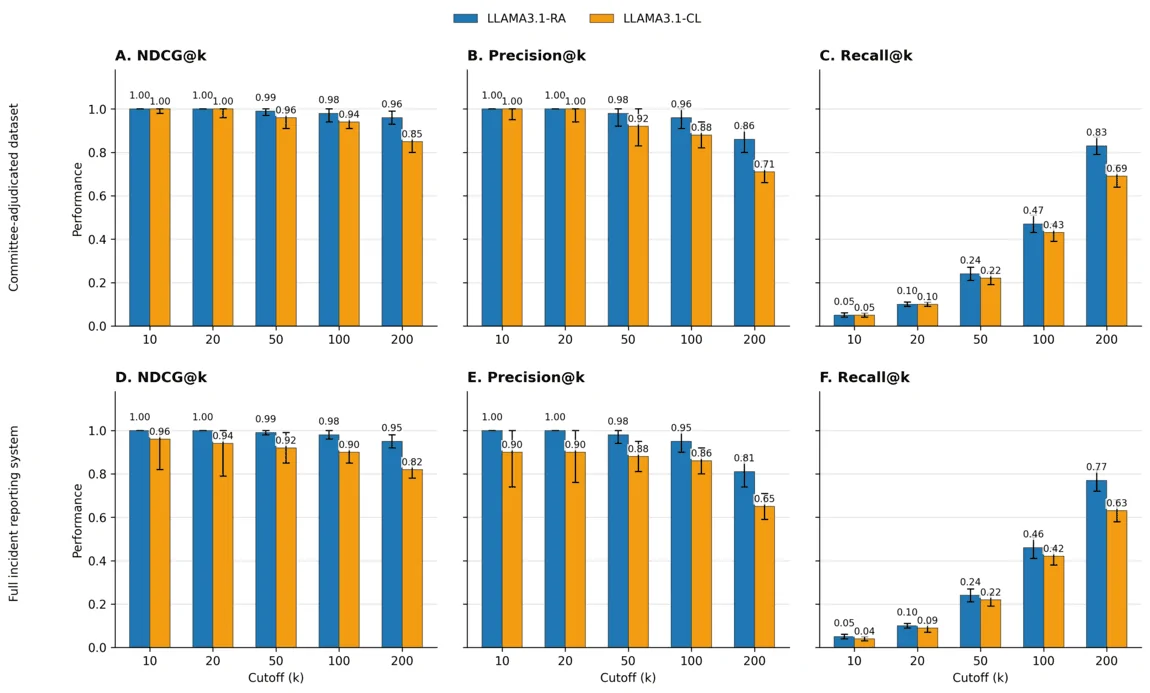
Performance comparison of the best-performing classification model (LLAMA3.1-CL) and ranking model (LLAMA3.1-RA) across cutoff values (k=10, 20, 50, 100, 200). (A-C) NDCG@k, Precision@k, and Recall@k for the committee-adjudicated dataset. (D-F) The corresponding metrics for the full incident reporting system dataset.

### Comparison With the Reporter-Assigned Severity Baseline

Table 3 presents the performance comparison between the best-performing ranking model (LLAMA3.1-RA) and the reporter-assigned severity baseline across both evaluation settings, while Figure 4 visualizes their NDCG@k, Precision@k, and Recall@k across the evaluated cutoff values. In panel A (committee-adjudicated dataset), LLAMA3.1-RA consistently achieved significantly higher mean performance than the baseline across all 16 evaluation metrics (all *P*<.001). Average precision was substantially higher for LLAMA3.1-RA (mean 0.94, 95% CI 0.90-0.98) compared with the baseline (mean 0.45, 95% CI 0.38-0.52), corresponding to a 108.9% relative improvement. Performance gains were evident even at the most restrictive cutoffs. For example, NDCG@10 and NDCG@20 improved by 31.6% and 37.0%, respectively, while Precision@10 and Precision@20 improved by 49.3% and 66.7%. At k=100, LLAMA3.1-RA demonstrated relative improvements of 58.1% in NDCG, 118.2% in Precision, and 123.8% in Recall. At k=200, improvements remained substantial, reaching 57.4% in NDCG, 104.8% in Precision, and 102.4% in Recall. Notably, the ranking model retrieved 83% of high-severity PSEs within the top 200 positions, compared with 41% under the reporter-assigned severity baseline.

In panel B (full IRS dataset), which reflects real-world class imbalance and operationally representative conditions, the superiority of LLAMA3.1-RA was even more pronounced. Average precision improved from 0.27 (95% CI 0.23-0.31) under the baseline to 0.89 (95% CI 0.84-0.94) with LLAMA3.1-RA, corresponding to a 229.6% relative improvement. Substantial gains were observed across all evaluation cutoffs. At k=100, relative improvements reached 172.2% for NDCG, 239.3% for precision, and 253.8% for recall. At k=200, LLAMA3.1-RA achieved improvements of 187.9% in NDCG, 237.5% in precision, and 266.7% in recall. These results indicate that, under operationally representative conditions with a low prevalence of HSEs, reliance on reporter-assigned severity alone performs markedly worse than the ranking-based approach, whereas LLAMA3.1-RA maintains robust prioritization performance.

**Table 3:** Comparative performance of the best-performing ranking model (LLAMA3.1-RA) and the reporter-assigned severity baseline across 2 evaluation settings: (panel A) the committee-adjudicated dataset, and (panel B) the full incident reporting system dataset reflecting real-world operationally representative conditions. Performance values are reported as mean (95% CI) on the held-out testing set, estimated using 2000 bootstrap resamples. Relative improvement (%) represents the proportional performance gain of LLAMA3.1-RA over the reporter-assigned severity baseline. *P* values indicate statistical significance based on paired comparisons with Bonferroni correction.

Metrics			Reporter-assigned severity baseline, mean (95% CI)		LLAMA3.1-RA, mean (95% CI)		Relative improvement (%)		*P* value		
Panel A. Committee-adjudicated dataset											
	Average precision	0.45 (0.38-0.52)		0.94 (0.90-0.98)		108.9		<.001			
	NDCG@10	0.76 (0.57-0.95)		1.00 (1.00-1.00)		31.6		<.001			
	NDCG@20	0.73 (0.56-0.90)		1.00 (0.99-1.00)		37.0		<.001			
	NDCG@50	0.65 (0.54-0.76)		0.99 (0.97-1.00)		52.3		<.001			
	NDCG@100	0.62 (0.54-0.70)		0.98 (0.94-1.00)		58.1		<.001			
	NDCG@200	0.61 (0.55-0.67)		0.96 (0.93-0.99)		57.4		<.001			
	Precision@10	0.67 (0.42-0.92)		1.00 (1.00-1.00)		49.3		<.001			
	Precision@20	0.60 (0.37-0.83)		1.00 (0.98-1.00)		66.7		<.001			
	Precision@50	0.48 (0.34-0.62)		0.98 (0.92-1.00)		104.2		<.001			
	Precision@100	0.44 (0.34-0.54)		0.96 (0.91-1.00)		118.2		<.001			
	Precision@200	0.42 (0.35-0.49)		0.86 (0.80-0.92)		104.8		<.001			
	Recall@10	0.03 (0.02-0.04)		0.05 (0.04-0.06)		66.7		<.001			
	Recall@20	0.06 (0.04-0.08)		0.10 (0.09-0.11)		66.7		<.001			
	Recall@50	0.12 (0.09-0.15)		0.24 (0.21-0.27)		100.0		<.001			
	Recall@100	0.21 (0.17-0.25)		0.47 (0.43-0.51)		123.8		<.001			
	Recall@200	0.41 (0.36-0.46)		0.83 (0.79-0.87)		102.4		<.001			
Panel B. Full incident reporting system dataset											
	Average precision	0.27 (0.23-0.31)		0.89 (0.84-0.94)		229.6		<.001			
	NDCG@10	0.46 (0.30-0.62)		1.00 (1.00-1.00)		117.4		<.001			
	NDCG@20	0.44 (0.29-0.59)		1.00 (0.99-1.00)		127.3		<.001			
	NDCG@50	0.41 (0.28-0.54)		0.99 (0.98-1.00)		141.5		<.001			
	NDCG@100	0.36 (0.28-0.44)		0.98 (0.96-1.00)		172.2		<.001			
	NDCG@200	0.33 (0.28-0.38)		0.95 (0.92-0.98)		187.9		<.001			
	Precision@10	0.45 (0.14-0.76)		1.00 (1.00-1.00)		122.2		<.001			
	Precision@20	0.44 (0.23-0.65)		1.00 (0.98-1.00)		127.3		<.001			
	Precision@50	0.38 (0.25-0.51)		0.98 (0.94-1.00)		157.9		<.001			
	Precision@100	0.28 (0.20-0.36)		0.95 (0.90-1.00)		239.3		<.001			
	Precision@200	0.24 (0.18-0.30)		0.81 (0.74-0.88)		237.5		<.001			
	Recall@10	0.02 (0.01-0.03)		0.05 (0.04-0.06)		150.0		<.001			
	Recall@20	0.04 (0.02-0.06)		0.10 (0.09-0.11)		150.0		<.001			
	Recall@50	0.08 (0.05-0.11)		0.24 (0.21-0.27)		200.0		<.001			
	Recall@100	0.13 (0.09-0.17)		0.46 (0.41-0.51)		253.8		<.001			
	Recall@200	0.21 (0.16-0.26)		0.77 (0.72-0.82)		266.7		<.001			

**Figure 4:**
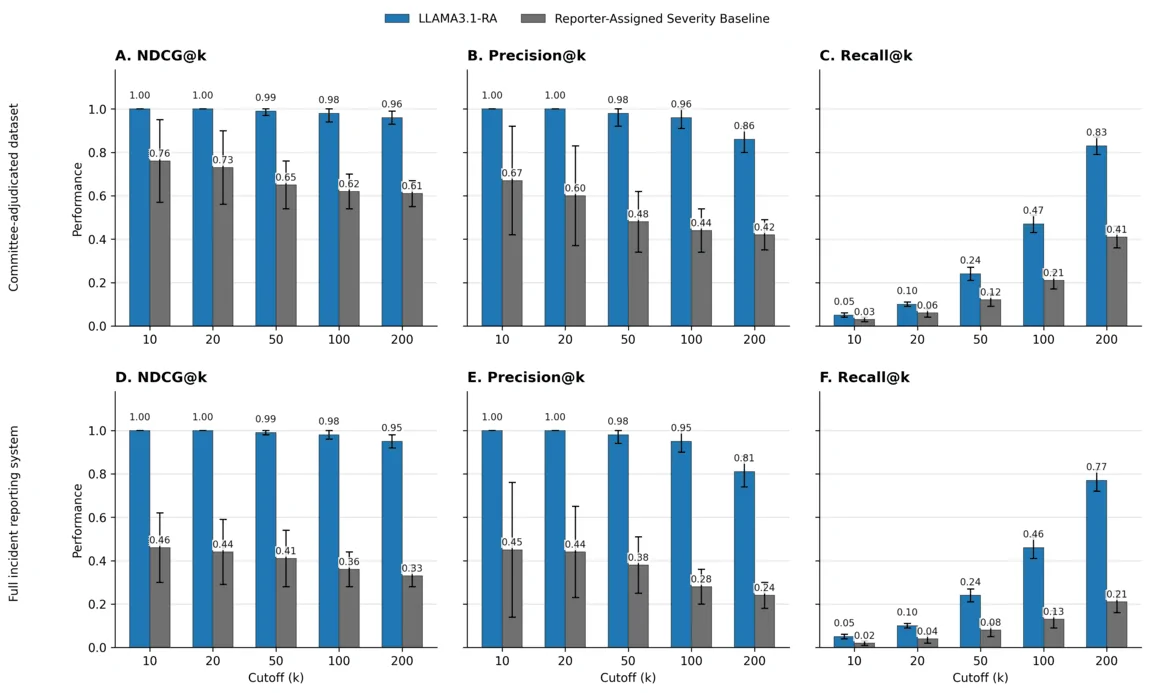
Performance comparison of the best-performing ranking model (LLAMA3.1-RA) and the reporter-assigned severity baseline across cutoff values (k=10, 20, 50, 100, 200). (A-C) NDCG@k, Precision@k, and Recall@k for the committee-adjudicated dataset. (D-F) The corresponding metrics for the full incident reporting system dataset.

## Discussion

### Overview

Adverse events remain a major threat to patient safety and impose substantial burdens on health care systems worldwide [6]. Although many countries have established mandatory reporting legislation [7-9], only a small proportion of PSEs undergo a comprehensive investigation due to the resource-intensive nature of the review process. Patient safety specialists are therefore responsible for manually triaging reported events to identify high-severity cases that warrant institutional investigation [18,23,24]. However, the rapidly increasing volume of reports, while reflecting ongoing efforts to strengthen reporting and safety culture, has rendered manual triage increasingly challenging [25]. Previous studies have applied NLP techniques to facilitate this process; nevertheless, most have relied on classification-based approaches [18,26,27,51], which may not fully align with the operational needs of patient safety specialists who must efficiently prioritize the most critical events requiring investigation. In contrast, ranking-based approaches learn the relative ordering of safety events by severity, making them particularly well-suited for this prioritization task. By directly optimizing the prioritization step within existing triage workflows, ranking-based models offer a practical and workflow-aligned advancement. To the best of our knowledge, this study is the first to develop and validate ranking-based models for prioritizing high-severity PSEs.

### Principal Results

The best-performing ranking model (LLAMA3.1-RA) demonstrated a significant advantage over both classification models and the reporter-assigned severity baseline in prioritizing high-severity PSEs. Compared with the best-performing classification model (LLAMA3.1-CL), LLAMA3.1-RA achieved a 22.1% relative improvement in average precision and significantly outperformed it in 10 of 16 evaluation metrics. Although both models achieved perfect NDCG and precision at the top 10 and top 20 ranked positions, LLAMA3.1-RA showed progressively greater gains at broader cutoffs, with relative improvements of 12.9% in NDCG@200, 21.1% in Precision@200, and 20.3% in Recall@200. These findings indicate that while both models effectively identified the most severe cases, the ranking-based approach maintained superior prioritization as the evaluation range expanded. Importantly, similar patterns were observed in the full IRS dataset (panel B), which reflects real-world class imbalance and operationally representative conditions. Despite the substantially lower prevalence of high-severity PSEs, LLAMA3.1-RA consistently outperformed the classification model across evaluation cutoffs. This consistency across both expert-adjudicated and operationally-representative datasets supports the robustness of the ranking-based approach and underscores its potential applicability within this study setting. Consequently, ranking-based approaches may provide a scalable solution for supporting triage workflows, particularly given the increasing volume of PSE reports in modern health care systems [52-54].

Compared with the reporter-assigned severity baseline, the LLAMA3.1-RA model demonstrated significantly superior capability in prioritizing high-severity PSEs across all 16 evaluation metrics. At the 200-event evaluation cutoff, LLAMA3.1-RA retrieved 83.0% of all high-severity PSEs, whereas the reporter-assigned severity baseline identified only 41.0%, corresponding to a 102.4% relative improvement. This substantial gain highlights the model’s ability to consistently detect critical events that may otherwise not be prioritized for timely review. The comparatively lower performance of the baseline likely reflects the inherent limitations of the reporter-assigned severity baseline, which is based on reporters’ subjective severity ratings that tend to be inconsistent due to variations in professional role, clinical experience, and familiarity with severity taxonomies [21,55]. Integrating ranking-based models such as LLAMA3.1-RA into institutional triage workflows may therefore enhance the efficiency and accuracy of prioritizing high-severity PSEs, enabling more timely identification and mitigation of such events, facilitating earlier escalation to leadership for timely organizational response, and ultimately strengthening patient safety outcomes.

In this study, the primary objective was to prioritize HSE reports for comprehensive institutional investigation within settings where review capacity is limited. This objective reflects the importance of directing available investigative resources toward events involving substantial patient harm. Alternative formulations may capture different aspects of the patient safety review process. For example, combining HSE and LSE could support the identification of events that reached the patient, while distinguishing escalated from nonescalated reports would model historical escalation decisions under existing institutional workflows. In comparison, the present formulation trains the models using committee-adjudicated severity labels to learn patterns associated with final assessed severity and prioritize incoming reports accordingly. The proposed models are intended to support report prioritization and potential escalation rather than automatically assign final severity labels. Future studies could compare these complementary formulations across different institutional review settings.

Although numerous studies have investigated the application of machine learning models to assist in the severity triage of safety events, few have proposed concrete designs or demonstrated how such technologies can be effectively embedded within clinical workflows [18,26,27,51]. Severity triage is an inherently high-stakes process, as decisions directly influence institutional response and allocation of resources for organizational improvements. Within this context, machine learning models should function as decision-support tools rather than autonomous decision-makers, and final judgment must remain a human decision to ensure ethical appropriateness [56-58]. In this study, the proposed ranking models are intended to support specialists by ordering incoming reports according to predicted severity, thereby facilitating the prioritization of reports for further review within available investigative capacity.

HSE prioritization was selected because HSEs represent the reports of greatest concern for comprehensive institutional investigation, particularly when review capacity is limited. Combining HSE and LSE would treat events involving substantial harm and those involving limited or no clinically significant harm as equally relevant. Moreover, escalation decisions are made during preliminary screening and may not reflect the final severity determined through multidisciplinary committee adjudication. The proposed models are therefore intended to assist the escalation process by ranking incoming reports according to predicted severity, while final escalation and severity determinations remain the responsibility of patient safety specialists and the multidisciplinary committee.

Although the proposed approach demonstrated consistent performance across the 2 evaluation settings, both datasets originated from the same health system. The learned ranking may therefore reflect institution-specific characteristics, including local reporting practices, incident descriptions, severity-assessment procedures, and triage workflows. Differences in these characteristics may influence model performance when applied in other settings. Accordingly, independent external validation is needed to determine the extent to which the proposed approach generalizes beyond the institutional context in which it was developed.

A related consideration in interpreting performance on the full IRS dataset is the operational treatment of reports that were not escalated for committee review. These reports were treated as NSEs because they had been considered lower priority within the existing institutional review workflow. This operational definition enabled evaluation under the class distribution encountered in routine practice; however, nonescalation does not independently confirm the absence of an HSE. Some genuinely severe events may not have been escalated because of incomplete information at submission, differences in reviewers’ experience or judgment, inconsistent application of escalation criteria, or human error during initial screening. Accordingly, the full IRS dataset may contain label noise if some nonescalated reports would have been classified as HSEs had they undergone multidisciplinary committee review.

The use of operational escalation status also means that historical triage decisions are incorporated into the supervision and evaluation signals. As a result, the models may learn not only textual patterns associated with adjudicated severity but also patterns reflecting existing institutional reporting and escalation practices. Performance on the full IRS dataset may therefore partly represent the model’s ability to reproduce or align with those practices rather than its ability to identify true event severity independently. This distinction is especially important because historical decisions may reflect institution-specific workflows, reporting cultures, resource constraints, and variation in how reports were documented and reviewed. Consequently, results from the full IRS dataset should be interpreted as evidence of performance under the study institution’s operational labeling framework, whereas the committee-adjudicated dataset provides the more direct assessment against expert-reviewed severity labels. Future studies could independently review a sample of nonescalated reports to estimate potential misclassification and evaluate whether model-assisted prioritization identifies severe events that were not captured through the historical escalation process.

### Limitations

This study has several limitations. First, it was conducted using data from a single academic health system in Canada, which may limit the generalizability of the findings to other institutional or geographical contexts. Future multicenter studies involving more diverse patient populations and reporting systems are warranted to enhance external validity. Second, the proposed model was evaluated retrospectively, and its prospective performance in real-world clinical settings remains to be determined. Future research should examine the model’s implementation feasibility and its influence on triage outcomes in operational environments. Third, the study dataset covered an extended period during which medical technologies, organizational structures, and incident-reporting practices may have evolved. Because the extracted data did not include detailed information regarding the timing or nature of specific technological, organizational, or reporting-system changes, we could not directly determine whether or how these developments influenced report content or writing style. As a related assessment of temporal stability, temporal validation demonstrated that LLAMA3.1-RA maintained broadly stable performance when trained on earlier reports and evaluated on more recent reports. However, this analysis does not directly establish whether the nature or writing of the reports changed over time. Future studies should directly examine changes in report characteristics and documentation practices across successive collection periods. Fourth, this study relied exclusively on IRS data and did not incorporate information from other relevant sources such as electronic medical records, maintenance and facility reports, or audit data, which patient safety specialists often use to validate and contextualize reported events. Future research could examine how similar prioritization approaches might integrate these data sources to complement the proposed AI-assisted triage workflow. Future research could also explore the application of ranking models directly to EMR systems, which may enable earlier identification of high-risk events and reduce reliance on downstream incident reporting workflows. In addition, future studies could analyze system-level and longitudinal trends in safety events to enable further automation and provide deeper insight into the underlying contributors to adverse events. Fifth, this study did not examine patient preferences or perspectives on the use of artificial intelligence to support incident severity prioritization. Future research should explicitly address these dimensions, particularly in relation to ethical, governance, and patient-centered considerations. Finally, although this study evaluated 2 open-source general-purpose large language models within a secure computing environment, it did not assess proprietary API-based models such as ChatGPT because the patient safety data could not be transmitted to external services. Future studies conducted within secure, institutionally governed environments could examine these additional models.

### Conclusion

This study developed and validated ranking-based machine learning models to prioritize high-severity PSEs. The proposed LLAMA3.1-RA model outperformed both classification-based models and the reporter-assigned severity baseline, demonstrating superior capability in prioritizing critical events requiring institutional investigation. The proposed ranking approach may support patient safety specialists in prioritizing incoming reports for further review, although prospective evaluation is required to determine its effectiveness within operational triage workflows. Collectively, this model-assisted triage framework offers a scalable and efficient solution for prioritizing high-severity PSEs, with the potential to enhance decision-making accuracy, workflow efficiency, and ultimately strengthen patient safety outcomes.

## Appendix

Multimedia Appendix 1 Synthetic examples of patient safety event reports and their severity determination.

Multimedia Appendix 2 Sensitivity analysis of multiclass and binary high-severity event classification formulations.

Multimedia Appendix 3 Summary of evaluated models and abbreviations, and feature extraction procedures for feature-based models.

Multimedia Appendix 4 Hyperparameter settings, tuning ranges, and final selected configurations for feature-based and transformer-based models.

Multimedia Appendix 5 Class-imbalance mitigation strategies and parameter settings.

Multimedia Appendix 6 Mathematical definitions of evaluation metrics.

Multimedia Appendix 7 Evaluation results of all developed ranking and classification models.

Multimedia Appendix 8 Performance of traditional classification baselines using bag-of-words and term frequency inverse document frequency representations.

Multimedia Appendix 9 Aggregate error and model-disagreement analysis.

Multimedia Appendix 10 Ablation analyses of training size and learning objective.

Multimedia Appendix 11 Temporal validation of the best-performing learning-to-rank model using chronologically partitioned patient safety event reports.

## Abbreviations

BAG: bag-of-words
HSE: high-severity event
IRS: incident reporting system
LSE: low-severity event
LTR: learning-to-rank
MNB: multinomial naïve Bayes
NDCG: normalized discounted cumulative gain
NLP: natural language processing
NME: near-miss event
NSE: no safety-concern event
PSE: patient safety event
SMOTE: Synthetic Minority Oversampling Technique
SVM: support vector machine
TF-IDF: term frequency inverse document frequency

## Data Availability

The data used in this study consist of patient safety event reports containing sensitive and potentially identifiable clinical information. Due to ethical restrictions, privacy legislation, and institutional data governance policies, the dataset and trained models cannot be shared publicly. Access to deidentified data may be considered upon reasonable request to LBC, subject to approval by the participating institutions and research ethics boards.
