# Supplementary material for: Machine Learning to Prioritize High-Severity Patient Safety Events for Institutional Investigation: Algorithm Development and Validation Study
Source: JMIR Med Inform. 2026 Sep 29;14:e101393. doi: 10.2196/101393 (PMC13662446; doi:10.2196/101393)
Supplement: Supplementary file 1 [file medinform_v14i1e101393_app1.docx]

## Appendix-1 Synthetic Examples of Patient Safety Event Reports and Their Severity Determination.

Table 1 presents three synthetic patient safety event reports to illustrate how the severity determination process is applied across different event circumstances and outcomes. For each example, the assigned severity category is accompanied by a rationale explaining the factors supporting the determination, including whether the event reached the patient, whether patient harm occurred, and whether additional clinical intervention was required. Together, these examples provide context for understanding how patient safety events are differentiated among high-severity, low-severity, and near-miss categories. The synthetic examples were created exclusively for explanatory purposes and do not reproduce or paraphrase any reports from the study datasets.

Table 1. Synthetic examples illustrating the determination of patient safety event severity.

| **Synthetic Incident Description** | **Severity Category** | **Rationale for Severity Determination** |
| --- | --- | --- |
| “The patient received twice the prescribed insulin dose and subsequently developed severe hypoglycemia with loss of consciousness, requiring emergency treatment and transfer to the intensive care unit.” | High-Severity Event | The medication error reached the patient and resulted in a life-threatening clinical outcome requiring emergency treatment and escalation to intensive care. |
| “The patient received twice the prescribed insulin dose. Blood glucose was monitored, and no symptoms or additional treatment were required.” | Low-Severity Event | Although the medication error reached the patient, no resulting harm or additional treatment was documented. |
| “A medication intended for another patient was brought to the bedside, but the discrepancy was identified before administration.” | Near-Miss Event | The error was identified before the medication reached the patient, and the event was therefore classified as a near miss. |
