# Supplementary material for: Machine Learning to Prioritize High-Severity Patient Safety Events for Institutional Investigation: Algorithm Development and Validation Study
Source: JMIR Med Inform. 2026 Sep 29;14:e101393. doi: 10.2196/101393 (PMC13662446; doi:10.2196/101393)
Supplement: Supplementary file 2 [file medinform_v14i1e101393_app10.docx]

## Appendix-10 Ablation Analyses of Training Size and Learning Objective.

To examine the effect of training-set size on ranking performance, we conducted an ablation analysis using LLAMA3.1-RA, the best-performing ranking model, and the committee-adjudicated dataset. The hyperparameter configuration selected for the final LLAMA3.1-RA model was held fixed across all experimental conditions. The original validation and testing partitions were also kept unchanged, while stratified subsets corresponding to 20%, 40%, 60%, 80%, and 100% of the original training partition were used for model training. The same preprocessing and training objective were applied in every condition so that differences in performance primarily reflected the amount of training data available. Table 1 reports Average Precision, NDCG@k, Precision@k, and Recall@k across all evaluated cutoff values for each training-size condition.

As shown in Table 1 and Figure 1, performance generally improved as the proportion of training data increased. Average Precision increased from 0.53 with 20% of the training partition to 0.94 with the complete training partition. Similar improvements were observed across NDCG, precision, and recall, with the effect of training size becoming particularly evident at broader evaluation cutoffs. At k = 200, NDCG increased from 0.67 to 0.96, precision increased from 0.48 to 0.86, and recall increased from 0.46 to 0.83 between the 20% and 100% conditions. At the more restrictive cutoffs, performance approached its maximum earlier; for example, NDCG@10 and Precision@10 reached 0.99 and 1.00, respectively, using 80% of the training data. The largest improvements generally occurred between the 20% and 80% conditions, followed by smaller gains between 80% and 100%, indicating that performance began to plateau as the complete training partition was approached. Nevertheless, the full training partition achieved the highest performance across nearly all evaluation metrics, demonstrating that LLAMA3.1-RA continued to benefit from additional adjudicated training reports.

Table 1. Effect of training-set size on the performance of LLAMA3.1-RA in the committee-adjudicated dataset.

| **Metric** | **20%**  **(n=374)** | **40%**  **(n=749)** | **60%**  **(n=1,123)** | **80%**  **(n=1,498)** | **100%**  **(n=1,872)** |
| --- | --- | --- | --- | --- | --- |
| Average Precision | 0.53 | 0.68 | 0.81 | 0.90 | 0.94 |
| NDCG@10 | 0.76 | 0.86 | 0.94 | 0.99 | 1.00 |
| NDCG@20 | 0.74 | 0.84 | 0.93 | 0.98 | 1.00 |
| NDCG@50 | 0.71 | 0.82 | 0.91 | 0.96 | 0.99 |
| NDCG@100 | 0.69 | 0.80 | 0.89 | 0.95 | 0.98 |
| NDCG@200 | 0.67 | 0.78 | 0.87 | 0.93 | 0.96 |
| Precision@10 | 0.70 | 0.80 | 0.90 | 1.00 | 1.00 |
| Precision@20 | 0.65 | 0.75 | 0.85 | 0.95 | 1.00 |
| Precision@50 | 0.60 | 0.70 | 0.82 | 0.92 | 0.98 |
| Precision@100 | 0.55 | 0.65 | 0.78 | 0.89 | 0.96 |
| Precision@200 | 0.48 | 0.61 | 0.73 | 0.82 | 0.86 |
| Recall@10 | 0.03 | 0.04 | 0.04 | 0.05 | 0.05 |
| Recall@20 | 0.06 | 0.07 | 0.08 | 0.09 | 0.10 |
| Recall@50 | 0.14 | 0.17 | 0.20 | 0.22 | 0.24 |
| Recall@100 | 0.27 | 0.31 | 0.38 | 0.43 | 0.47 |
| Recall@200 | 0.46 | 0.59 | 0.71 | 0.79 | 0.83 |

Figure 1. Effect of training-set size on LLAMA3.1-RA performance in the committee-adjudicated dataset

Table 2 presents the impact of the learning objective on LLAMA3.1 performance. Cross-entropy, LambdaRank, and ListNet achieved identical NDCG, precision, and recall at k = 10 and k = 20, indicating comparable performance at the most restrictive evaluation cutoffs. Differences became more pronounced as the evaluation range expanded. LambdaRank achieved the highest Average Precision of 0.94, compared with 0.89 for ListNet and 0.77 for cross-entropy. At k = 200, LambdaRank achieved an NDCG of 0.96, a precision of 0.86, and a recall of 0.83. The corresponding values were 0.90, 0.79, and 0.76 for ListNet and 0.85, 0.71, and 0.69 for cross-entropy. These findings indicate that the learning objective had limited influence at the most restrictive cutoffs but became increasingly important as more reports were considered. Overall, LambdaRank provided the most consistent prioritization across the broader ranked list, while ListNet produced intermediate performance between LambdaRank and cross-entropy.

Table 2. Effect of learning objective on LLAMA3.1 performance in the committee-adjudicated dataset.

| **Metric** | **Cross-entropy** | **Lambdarank** | **Listnet** |
| --- | --- | --- | --- |
| Average Precision | 0.77 | 0.94 | 0.89 |
| NDCG@10 | 1.00 | 1.00 | 1.00 |
| NDCG@20 | 1.00 | 1.00 | 1.00 |
| NDCG@50 | 0.96 | 0.99 | 0.98 |
| NDCG@100 | 0.94 | 0.98 | 0.96 |
| NDCG@200 | 0.85 | 0.96 | 0.90 |
| Precision@10 | 1.00 | 1.00 | 1.00 |
| Precision@20 | 1.00 | 1.00 | 1.00 |
| Precision@50 | 0.92 | 0.98 | 0.96 |
| Precision@100 | 0.88 | 0.96 | 0.92 |
| Precision@200 | 0.71 | 0.86 | 0.79 |
| Recall@10 | 0.05 | 0.05 | 0.05 |
| Recall@20 | 0.10 | 0.10 | 0.10 |
| Recall@50 | 0.22 | 0.24 | 0.23 |
| Recall@100 | 0.43 | 0.47 | 0.44 |
| Recall@200 | 0.69 | 0.83 | 0.76 |
