# Supplementary material for: Machine Learning to Prioritize High-Severity Patient Safety Events for Institutional Investigation: Algorithm Development and Validation Study
Source: JMIR Med Inform. 2026 Sep 29;14:e101393. doi: 10.2196/101393 (PMC13662446; doi:10.2196/101393)
Supplement: Supplementary file 3 [file medinform_v14i1e101393_app11.docx]

## Appendix-11 Temporal Validation of the Best-Performing Learning-to-Rank Model Using Chronologically Partitioned Patient Safety Event Reports.

To evaluate whether the proposed ranking approach remained effective when applied to reports submitted after the model-development period, we conducted a temporal validation analysis using LLAMA3.1-RA, the best-performing ranking model. Reports in the committee-adjudicated dataset were ordered chronologically according to their submission dates. The earliest 70% of reports were used for training, the subsequent 10% were used for validation, and the most recent 20% were reserved for testing. This chronological partitioning approximated prospective implementation by evaluating whether patterns learned from earlier reports generalized to reports submitted during a later period.

The preprocessing procedures, learning objective, candidate hyperparameter ranges, and evaluation metrics were consistent with those used in the primary analysis. Model selection and hyperparameter optimization were performed using only the chronologically defined training and validation partitions, and the most recent testing partition was not used during model development. The final hyperparameter configuration was selected based on validation Average Precision. Table 1 presents the performance obtained under chronological partitioning, with the results from the primary random-split analysis included for descriptive comparison.

LLAMA3.1-RA maintained strong performance under chronological partitioning. Average Precision was 0.92, compared with 0.94 in the primary random-split analysis. Performance at the most restrictive cutoffs was nearly identical under the two partitioning strategies. At k = 200, NDCG was 0.94 under chronological partitioning compared with 0.96 under random partitioning, while precision was 0.84 compared with 0.86 and recall was 0.81 compared with 0.83. Similar modest differences were observed across the remaining cutoff values.

Overall, these findings indicate that LLAMA3.1-RA maintained broadly stable prioritization performance when trained on earlier reports and evaluated on reports submitted during a later period. This provides evidence of temporal robustness across the study period, with only modest differences observed when the model was evaluated on reports submitted during a later period. However, because the dataset did not document the timing or nature of specific technological, organizational, or reporting-system changes, this analysis cannot determine whether particular changes occurred or attribute the observed performance differences to individual temporal factors.

Table 1. Performance of LLAMA3.1-RA under random and chronological data-partitioning strategies in the committee-adjudicated dataset.

| **Metric** | **Primary random split** | **Temporal split** |
| --- | --- | --- |
| Average Precision | 0.94 | 0.92 |
| NDCG@10 | 1.00 | 1.00 |
| NDCG@20 | 1.00 | 0.99 |
| NDCG@50 | 0.99 | 0.98 |
| NDCG@100 | 0.98 | 0.97 |
| NDCG@200 | 0.96 | 0.94 |
| Precision@10 | 1.00 | 1.00 |
| Precision@20 | 1.00 | 0.95 |
| Precision@50 | 0.98 | 0.96 |
| Precision@100 | 0.96 | 0.93 |
| Precision@200 | 0.86 | 0.84 |
| Recall@10 | 0.05 | 0.05 |
| Recall@20 | 0.10 | 0.09 |
| Recall@50 | 0.24 | 0.23 |
| Recall@100 | 0.47 | 0.45 |
| Recall@200 | 0.83 | 0.81 |
