# Supplementary material for: Machine Learning to Prioritize High-Severity Patient Safety Events for Institutional Investigation: Algorithm Development and Validation Study
Source: JMIR Med Inform. 2026 Sep 29;14:e101393. doi: 10.2196/101393 (PMC13662446; doi:10.2196/101393)
Supplement: Supplementary file 4 [file medinform_v14i1e101393_app2.docx]

## Appendix-2 Sensitivity Analysis of Multiclass and Binary HSE Classification Formulations.

To examine whether the multiclass outcome formulation influenced the comparison between classification and ranking, we conducted a targeted sensitivity analysis using LLAMA3.1, the best-performing classification architecture. In the primary multiclass formulation, LLAMA3.1-CL was trained to predict the original HSE, LSE, NME, and NSE categories, and reports were ordered according to their predicted probabilities of receiving the HSE label. For the binary formulation, the original labels were collapsed into HSE and non-HSE categories, with LSE, NME, and NSE reports assigned to the non-HSE category. The resulting LLAMA3.1-CL-Binary model was trained to distinguish HSE from non-HSE reports directly, and reports were ordered according to their predicted probabilities of belonging to the HSE class.

The original training, validation, and testing partitions were retained. The binary model underwent the same hyperparameter-selection procedure as the multiclass model, using the unchanged validation set and the same candidate ranges for learning rate, batch size, number of training epochs, warmup ratio, and weight decay. Class imbalance was addressed using class-weighted cross-entropy loss, with weights calculated from the HSE and non-HSE frequencies in the binary training partition. The final hyperparameter configuration was selected based on validation Average Precision. No oversampling was applied to the transformer-based binary model, consistent with the imbalance-mitigation procedure used for the primary multiclass transformer-based classification models.

As shown in Table 1, all three formulations achieved identical performance at the most restrictive cutoffs, with NDCG and precision of 1.00 at k = 10 and k = 20. Differences emerged as the evaluation range expanded. LLAMA3.1-CL-Binary achieved an Average Precision of 0.79, compared with 0.77 for LLAMA3.1-CL. At k = 200, the binary formulation increased precision from 0.71 to 0.77 and recall from 0.69 to 0.74, indicating that directly optimizing the distinction between HSE and non-HSE reports provided a modest improvement in HSE-focused retrieval relative to multiclass classification. Despite its improved HSE-focused retrieval, LLAMA3.1-CL-Binary achieved lower NDCG than LLAMA3.1-CL at the broader cutoffs. At k = 200, NDCG was 0.82 for LLAMA3.1-CL-Binary and 0.85 for LLAMA3.1-CL. This pattern is consistent with the difference between the two outcome formulations. The binary model was trained to distinguish HSE from non-HSE reports but did not explicitly learn distinctions among LSE, NME, and NSE reports. In contrast, the multiclass model retained information about the complete severity structure during training, resulting in stronger graded ordering despite retrieving fewer HSE reports.

LLAMA3.1-RA continued to achieve the highest performance overall, with an Average Precision of 0.94 and NDCG, precision, and recall of 0.96, 0.86, and 0.83, respectively, at k = 200. LLAMA3.1-CL-Binary achieved an Average Precision of 0.79 and NDCG@200, Precision@200, and Recall@200 values of 0.82, 0.77, and 0.74, respectively. These findings demonstrate that the performance advantage of LLAMA3.1-RA was not attributable solely to the four-category outcome formulation used for the primary classification comparator. Although binary HSE classification improved HSE-focused retrieval relative to multiclass classification, LLAMA3.1-RA achieved higher performance than both classification formulations on Average Precision, Precision@k, Recall@k, and NDCG@k. These findings provide additional support for the ranking formulation as the strongest evaluated approach for both retrieving HSE reports and maintaining severity-aligned prioritization across the broader ranked list.

Table 1. Comparison of multiclass classification, binary HSE classification, and learning-to-rank formulations using LLAMA3.1 in the committee-adjudicated dataset.

| **Metric** | **LLAMA3.1-CL** | **LLAMA3.1-CL-Binary** | **LLAMA3.1-RA** |
| --- | --- | --- | --- |
| Average Precision | 0.77 | 0.79 | 0.94 |
| NDCG@10 | 1.00 | 1.00 | 1.00 |
| NDCG@20 | 1.00 | 1.00 | 1.00 |
| NDCG@50 | 0.96 | 0.95 | 0.99 |
| NDCG@100 | 0.94 | 0.90 | 0.98 |
| NDCG@200 | 0.85 | 0.82 | 0.96 |
| Precision@10 | 1.00 | 1.00 | 1.00 |
| Precision@20 | 1.00 | 1.00 | 1.00 |
| Precision@50 | 0.92 | 0.94 | 0.98 |
| Precision@100 | 0.88 | 0.92 | 0.96 |
| Precision@200 | 0.71 | 0.77 | 0.86 |
| Recall@10 | 0.05 | 0.05 | 0.05 |
| Recall@20 | 0.10 | 0.10 | 0.10 |
| Recall@50 | 0.22 | 0.23 | 0.24 |
| Recall@100 | 0.43 | 0.44 | 0.47 |
| Recall@200 | 0.69 | 0.74 | 0.83 |
