# Supplementary material for: Machine Learning to Prioritize High-Severity Patient Safety Events for Institutional Investigation: Algorithm Development and Validation Study
Source: JMIR Med Inform. 2026 Sep 29;14:e101393. doi: 10.2196/101393 (PMC13662446; doi:10.2196/101393)
Supplement: Supplementary file 5 [file medinform_v14i1e101393_app3.docx]

## Appendix-3 Summary of Evaluated Models and Abbreviations, and Feature Extraction Procedures for Feature-Based Models.

Table 1 summarizes the feature-based and transformer-based models evaluated in this study, together with their abbreviations and full model names. Models with the suffix “RA” were developed for ranking, whereas models with the suffix “CL” were developed for classification. This table is provided as a reference to support interpretation of the model abbreviations used throughout the manuscript.

Table 1. Summary of models used in this study, including their abbreviations and corresponding full model names. Models with the suffix “RA” were developed for ranking tasks, while those with the suffix “CL” were developed for classification tasks.

| **Task type** | **Abbreviations** | **Full model name** |
| --- | --- | --- |
| Ranking | XGB-RA | XGBoost |
|  | LGBM-RA | LightGBM |
|  | BERT-RA | bert-base-uncased |
|  | ROBERTA-RA | roberta-base |
|  | XLM-ROBERTA-RA | xlm-roberta-base |
|  | BIOBERT-RA | biobert-v1.1 |
|  | BIOMEDBERT-RA | BiomedNLP-BiomedBERT-base-uncased-abstract-fulltext |
|  | GATORTRON-RA | gatortron-base |
|  | LLAMA3.1-RA | Llama-3.1-8B |
|  | QWEN3-RA | Qwen3-8B-Base |
| Classification | XGB-CL | XGBoost |
|  | LGBM-CL | LightGBM |
|  | BERT-CL | bert-base-uncased |
|  | ROBERTA-CL | roberta-base |
|  | XLM-ROBERTA-CL | xlm-roberta-base |
|  | BIOBERT-CL | biobert-v1.1 |
|  | BIOMEDBERT-CL | BiomedNLP-BiomedBERT-base-uncased-abstract-fulltext |
|  | GATORTRON-CL | gatortron-base |
|  | LLAMA3.1-CL | Llama-3.1-8B |
|  | QWEN3-CL | Qwen3-8B-Base |

Feature-based models require data to be transformed into text representations before being used as model inputs. To this end, we extracted two categories of features from the incident descriptions: static and contextual text representations. Static representations are derived using traditional vectorization methods that often result in sparse, high-dimensional feature spaces and have limited ability to capture word order and semantic relationships [1]. In contrast, contextual representations capture the semantic meaning of words in relation to their surrounding text, using embeddings generated by pretrained transformer-based language models [2,3].

For static text representations, preprocessing is essential to ensure consistent input formatting and to enable the model to recognize words in different forms. We applied standard normalization techniques, including removing non-alphabetic characters and stop words, converting text to lowercase, and applying stemming to reduce words to their root forms. In contrast, contextual text representations do not require such preprocessing, as they are generated by transformer-based models pretrained on large-scale raw text corpora [3,4]. These models inherently account for linguistic variations such as plurality and tense.

A total of six distinct text-representation methods were used in this study (Table 2). Static and contextual representations were generated separately and were not concatenated or otherwise combined. Each BAG, TF-IDF, or transformer-derived representation was independently supplied to XGBoost and LightGBM, resulting in a distinct model–representation combination. Static representations were generated using bag-of-words (BAG) and term frequency–inverse document frequency (TF-IDF). BAG represented each report according to token occurrence counts, whereas TF-IDF weighted each token according to its frequency within the report and its relative rarity across the training corpus. Both methods incorporated n-gram features ranging from unigrams to trigrams. The vectorizers were fitted exclusively to the training data and subsequently applied to the validation and testing sets using the vocabulary learned from the training data.

Contextual representations were independently extracted using BERT, ROBERTA, BIOBERT, and GATORTRON [3–6]. Each incident description was processed using the tokenizer corresponding to the pretrained model and truncated or padded to a maximum sequence length of 512 tokens. Report-level embeddings were obtained from the final hidden-layer representation of the first sequence token, producing a fixed-dimensional vector for each report. The pretrained transformer parameters remained frozen during feature extraction.

Table 2. Static and contextual text representations used for developing feature-based models in this study. The suffix “_R” indicates a text representation derived from the corresponding method or model.

| **Representation categories** | **Full model name** | **Abbreviations** |
| --- | --- | --- |
| Static | Bag-of-words | BAG_R |
|  | Term Frequency Inverse Document Frequency | TF-IDF_R |
| Contextual | bert-base-uncased | BERT_R |
|  | roberta-base | ROBERTA_R |
|  | biobert-v1.1 | BIOBERT_R |
|  | gatortron-base | GATORTRON_R |

**References**

1.	Wang Y, Hou Y, Che W, Liu T. From static to dynamic word representations: a survey. Int J Mach Learn & Cyber 2020 Jul 1;11(7):1611–1630. doi: 10.1007/s13042-020-01069-8

2.	Liu Q, Kusner MJ, Blunsom P. A Survey on Contextual Embeddings. 2020 Mar 16; doi: 10.48550/arXiv.2003.07278

3.	Devlin J, Chang M-W, Lee K, Toutanova K. BERT: Pre-training of Deep Bidirectional Transformers for Language Understanding. Proceedings of the 2019 Conference of the North American Chapter of the Association for Computational Linguistics: Human Language Technologies, Volume 1 (Long and Short Papers) Minneapolis, Minnesota: Association for Computational Linguistics; 2019. p. 4171–4186. doi: 10.18653/v1/N19-1423

4.	Liu Y, Ott M, Goyal N, Du J, Joshi M, Chen D, Levy O, Lewis M, Zettlemoyer L, Stoyanov V. RoBERTa: A Robustly Optimized BERT Pretraining Approach. arXiv; 2019. doi: 10.48550/arXiv.1907.11692

5.	Yang X, Chen A, PourNejatian N, Shin HC, Smith KE, Parisien C, Compas C, Martin C, Flores MG, Zhang Y, Magoc T, Harle CA, Lipori G, Mitchell DA, Hogan WR, Shenkman EA, Bian J, Wu Y. GatorTron: A Large Clinical Language Model to Unlock Patient Information from Unstructured Electronic Health Records. arXiv; 2022. doi: 10.48550/arXiv.2203.03540

6.	Lee J, Yoon W, Kim S, Kim D, Kim S, So CH, Kang J. BioBERT: a pre-trained biomedical language representation model for biomedical text mining. Bioinformatics 2020 Feb 15;36(4):1234–1240. doi: 10.1093/bioinformatics/btz682
