# Supplementary material for: Machine Learning to Prioritize High-Severity Patient Safety Events for Institutional Investigation: Algorithm Development and Validation Study
Source: JMIR Med Inform. 2026 Sep 29;14:e101393. doi: 10.2196/101393 (PMC13662446; doi:10.2196/101393)
Supplement: Supplementary file 6 [file medinform_v14i1e101393_app4.docx]

## Appendix-4 Hyperparameter Settings, Tuning Ranges, and Final Selected Configurations for Feature-Based and Transformer-Based Models.

This appendix presents the hyperparameter settings and tuning ranges used during model development for both feature-based and transformer-based models. Tables 1–4 summarize the settings and tuning ranges for the feature-based models (XGB-RA, LGBM-RA, XGB-CL, and LGBM-CL). Tables 5 and 6 present the corresponding information for transformer-based ranking and classification models. Table 7 reports the final selected hyperparameter configuration for the best-performing model within each major model family.

Table 1. Hyperparameter settings and tuning ranges for the XGB-RA model (XGBoost for ranking).

| **Parameter Name** | **Description** | **Tuning Range or Fixed Value** |
| --- | --- | --- |
| objective | Specifies the learning objective and loss function used to optimize the ranking model. | [‘rank:ndcg’,  ‘rank:pairwise’] |
| lambdarank_pair_method | Defines the method used to construct item pairs for pairwise ranking. | [‘mean’] |
| lambdarank_num_pair_per_sample | Specifies the number of document pairs generated  for each document. | [5, 10] |
| learning_rate | Controls the contribution of each tree when added to the model. Smaller values improve generalization but require more boosting rounds. | [0.01, 0.05] |
| n_estimators | Total number of boosting rounds during model training. | [50, 150] |
| max_depth | Maximum depth of each decision tree. Increasing depth allows the model to capture more complex patterns. | [3, 6] |
| subsample | Fraction of training instances randomly sampled for each boosting iteration. Helps prevent overfitting. | [0.8, 1.0] |
| reg_alpha | L1 regularization term which encourages sparsity in the model and helps reduce overfitting. | [0, 1.0] |
| reg_lambda | L2 regularization term which penalizes large weights to help prevent overfitting. | [0, 1.0] |
| gamma | Minimum loss reduction required to make a further split on a leaf node. Helps prevent overfitting. | [0, 1.0] |

Table 2. Hyperparameter settings and tuning ranges for the LGBM-RA model (LightGBM for ranking).

| **Parameter Name** | **Description** | **Tuning Range or Fixed Value** |
| --- | --- | --- |
| objective | Specifies the learning objective and loss function used to optimize the ranking model. | [‘lambdarank’] |
| boosting_type | Specifies the type of boosting algorithm to use, which determines how new trees are added during training. | [‘gbdt’] |
| num_boost_round | Total number of boosting rounds during model training. | [50, 150] |
| learning_rate | Controls the contribution of each tree when added to the model. Smaller values improve generalization but require more boosting rounds. | [0.01, 0.05] |
| max_depth | Maximum depth of each decision tree. Increasing depth allows the model to capture more complex patterns. | [3, 6] |
| feature_fraction | Fraction of training features randomly sampled for each boosting iteration. Helps prevent overfitting. | [0.8, 1.0] |
| reg_alpha | L1 regularization term which encourages sparsity in the model and helps reduce overfitting. | [0, 1.0] |
| reg_lambda | L2 regularization term which penalizes large weights to help prevent overfitting. | [0, 1.0] |
| num_leaves | Maximum number of leaves in each tree; controls the complexity of the model. | [10, 25] |
| min_data_in_leaf | Minimum number of samples required in a leaf node. Larger values can prevent the model from creating overly specific leaf nodes, reducing overfitting. | [10, 20] |

Table 3. Hyperparameter settings and tuning ranges for the XGB-CL model (XGBoost for classification).

| **Parameter Name** | **Description** | **Tuning Range or Fixed Value** |
| --- | --- | --- |
| objective | Specifies the learning objective and loss function used to optimize the classification model. | [‘multi:softprob’] |
| learning_rate | Controls the contribution of each tree when added to the model. Smaller values improve generalization but require more boosting rounds. | [0.01, 0.05] |
| n_estimators | Total number of boosting rounds during model training. | [50, 150] |
| max_depth | Maximum depth of each decision tree. Increasing depth allows the model to capture more complex patterns. | [3, 6] |
| subsample | Fraction of training instances randomly sampled for each boosting iteration. Helps prevent overfitting. | [0.8, 1.0] |
| reg_alpha | L1 regularization term which encourages sparsity in the model and helps reduce overfitting. | [0, 1.0] |
| reg_lambda | L2 regularization term which penalizes large weights to help prevent overfitting. | [0, 1.0] |
| gamma | Minimum loss reduction required to make a further split on a leaf node. Helps prevent overfitting. | [0, 1.0] |

Table 4. Hyperparameter settings and tuning ranges for the LGBM-CL model (LightGBM for classification).

| **Parameter Name** | **Description** | **Tuning Range or Fixed Value** |
| --- | --- | --- |
| objective | Specifies the learning objective and loss function used to optimize the classification model. | [‘multiclass’] |
| boosting_type | Specifies the type of boosting algorithm to use, which determines how new trees are added during training. | [‘gbdt’] |
| num_boost_round | Total number of boosting rounds during model training. | [50, 150] |
| learning_rate | Controls the contribution of each tree when added to the model. Smaller values improve generalization but require more boosting rounds. | [0.01, 0.05] |
| max_depth | Maximum depth of each decision tree. Increasing depth allows the model to capture more complex patterns. | [3, 6] |
| feature_fraction | Fraction of training features randomly sampled for each boosting iteration. Helps prevent overfitting. | [0.8, 1.0] |
| reg_alpha | L1 regularization term which encourages sparsity in the model and helps reduce overfitting. | [0, 1.0] |
| reg_lambda | L2 regularization term which penalizes large weights to help prevent overfitting. | [0, 1.0] |
| num_leaves | Maximum number of leaves in each tree; controls the complexity of the model. | [10, 25] |
| min_data_in_leaf | Minimum number of samples required in a leaf node. Larger values can prevent the model from creating overly specific leaf nodes, reducing overfitting. | [10, 20] |

**Table 5.** Hyperparameter settings and tuning ranges for transformer-based ranking models.

| **Parameter Name** | **Description** | **Tuning Range or Fixed Value** |
| --- | --- | --- |
| objective | Specifies the learning objective and loss function used to optimize the ranking model. | [‘lambdarank’, ‘listnet’] |
| learning rate | Step size controlling how much the model parameters are updated at each optimization step. | [1e-5, 5e-5] |
| batch_size | Number of training examples processed together in a single forward/backward pass before updating model parameters. | [8, 16] |
| warmup_ratio | Fraction of the total training steps used to gradually increase the learning rate from zero to the target value, helping to stabilize early training. | [0.10] |
| num_train_epochs | Number of complete passes through the entire training dataset during model training. | [3, 5] |
| weight_decay | L2 regularization coefficient applied to model weights to reduce overfitting by penalizing large parameter values. | [0.01] |

Table 6. Hyperparameter settings and tuning ranges for transformer-based classification models.

| **Parameter Name** | **Description** | **Tuning Range or Fixed Value** |
| --- | --- | --- |
| objective | Specifies the learning objective and loss function used to optimize the classification model (e.g., cross-entropy loss for multi-class classification). | [‘Cross-entropy loss’] |
| learning rate | Step size controlling how much the model parameters are updated at each optimization step. | [1e-5, 5e-5] |
| batch_size | Number of training examples processed together in a single forward/backward pass before updating model parameters. | [8, 16] |
| warmup_ratio | Fraction of the total training steps used to gradually increase the learning rate from zero to the target value, helping to stabilize early training. | [0.10] |
| num_train_epochs | Number of complete passes through the entire training dataset during model training. | [3, 5] |
| weight_decay | L2 regularization coefficient applied to model weights to reduce overfitting by penalizing large parameter values. | [0.01] |

Table 7. Final selected hyperparameter configurations for the best-performing model–representation combination within each model family across the two evaluation datasets.

| **Model Family** | **Best-Performing Model** | **Final Selected Hyperparameters** |
| --- | --- | --- |
| **Panel A. Committee-adjudicated dataset** | | |
| Feature-based ranking (XGB-RA) | XGB-RA + GATORTRON | objective=rank:ndcg  lambdarank_pair_method=mean  lambdarank_num_pair_per_sample=10  learning_rate=0.01  n_estimators=150; max_depth=6  subsample=0.8  reg_alpha=0  reg_lambda=1.0  gamma=0 |
| Feature-based ranking (LGBM-RA) | LGBM-RA + GATORTRON | objective=lambdarank  boosting_type=gbdt  num_boost_round=50  learning_rate=0.05  max_depth=6  feature_fraction=0.8  reg_alpha=0  reg_lambda=1.0  num_leaves=25  min_data_in_leaf=20 |
| Feature-based classification (XGB-CL) | XGB-CL + BAG | objective=multi:softprob  learning_rate=0.01  n_estimators=150  max_depth=6  subsample=0.8  reg_alpha=1.0  reg_lambda=1.0  gamma=0 |
| Feature-based classification (LGBM-CL) | LGBM-CL + ROBERTA | objective=multiclass  boosting_type=gbdt  num_boost_round=150  learning_rate=0.01  max_depth=6  feature_fraction=0.8  reg_alpha=1.0  reg_lambda=1.0  num_leaves=25  min_data_in_leaf=20 |
| Transformer-based ranking | LLAMA3.1_RA | objective=lambdarank  learning_rate=5e-5  batch_size=8  warmup_ratio=0.10  num_train_epochs=3  weight_decay=0.01 |
| Transformer-based classification | LLAMA3.1_CL | objective=cross-entropy loss  learning_rate=1e-5  batch_size=8  warmup_ratio=0.10  num_train_epochs=3  weight_decay=0.01 |
| **Panel B. Full incident reporting system dataset** | | |
| Feature-based ranking (XGB-RA) | XGB-RA + GATORTRON | objective=rank:ndcg  lambdarank_pair_method=mean  lambdarank_num_pair_per_sample=10  learning_rate=0.05  n_estimators=150  max_depth=6  subsample=0.8  reg_alpha=1.0  reg_lambda=1.0  gamma=0 |
| Feature-based ranking (LGBM-RA) | LGBM-RA + GATORTRON | objective=lambdarank  boosting_type=gbdt  num_boost_round=150  learning_rate=0.01  max_depth=6  feature_fraction=1.0  reg_alpha=1.0  reg_lambda=1.0  num_leaves=25  min_data_in_leaf=20 |
| Feature-based classification (XGB-CL) | XGB-CL + BERT | objective=multi:softprob  learning_rate=0.05  n_estimators=150  max_depth=3  subsample=0.8  reg_alpha=1.0  reg_lambda=1.0  gamma=1.0 |
| Feature-based classification (LGBM-CL) | LGBM-CL + BERT | objective=multiclass  boosting_type=gbdt  num_boost_round=50  learning_rate=0.05  max_depth=6  feature_fraction=0.8  reg_alpha=1.0  reg_lambda=1.0  num_leaves=25  min_data_in_leaf=20 |
| Transformer-based ranking | LLAMA3.1_RA | objective=lambdarank  learning_rate=5e-5  batch_size=8  warmup_ratio=0.10  num_train_epochs=5  weight_decay=0.01 |
| Transformer-based classification | LLAMA3.1_CL | objective=cross-entropy loss  learning_rate=5e-5  batch_size=8  warmup_ratio=0.10  num_train_epochs=3  weight_decay=0.01 |
