# Supplementary material for: Machine Learning to Prioritize High-Severity Patient Safety Events for Institutional Investigation: Algorithm Development and Validation Study
Source: JMIR Med Inform. 2026 Sep 29;14:e101393. doi: 10.2196/101393 (PMC13662446; doi:10.2196/101393)
Supplement: Supplementary file 7 [file medinform_v14i1e101393_app5.docx]

## Appendix-5 Class-Imbalance Mitigation Strategies and Parameter Settings.

Tables 1–4 describe the class-imbalance mitigation procedures used for the feature-based and transformer-based classification models, including the resampling and loss-weighting parameters, the numbers of synthetic samples generated and NSE reports downsampled, and the final class weights used during transformer-based model training. All imbalance-mitigation procedures were restricted to the training data, while the original class distributions were preserved in the validation and testing sets.

Table 1 summarizes the resampling procedures used for the feature-based classification models. For the committee-adjudicated dataset, SMOTE was applied with five nearest neighbors to increase the number of LSE, NME, and NSE reports until each category matched the 830 HSE training reports. This procedure generated 166 LSE, 785 NME, and 229 NSE synthetic samples, for a total of 1,180. For the full incident reporting system dataset, SMOTE was first used to increase the LSE and NME categories to 830 samples each, generating 166 LSE and 785 NME synthetic samples, for a total of 951. Because NSE reports constituted the substantial majority of the full dataset, this category was subsequently downsampled without replacement.

Table 2 describes the class-imbalance mitigation procedures used for the transformer-based classification models. For the committee-adjudicated dataset, balanced class-weighted cross-entropy loss was used to increase the contribution of less frequent severity categories during training. For the full incident reporting system dataset, the majority NSE category was first downsampled without replacement, after which balanced class weights were calculated from the resulting training distribution. These weights were supplied through the weight parameter of torch.nn.CrossEntropyLoss and remained fixed throughout model training. Unlike the feature-based models, the transformer-based models did not use SMOTE.

Table 3 reports the extent of NSE downsampling for each model and text representation. Three configurations were evaluated, retaining one, one-and-a-half, or two NSE reports for every combined HSE, LSE, and NME report in the training set. The configuration yielding the highest validation Average Precision was selected separately for each model. For the feature-based models, this process retained 2,490–4,980 of the original 79,453 NSE training reports and removed 74,473–76,963 reports. For the transformer-based models, 1,346–2,019 of the original 69,521 NSE reports were retained, while 67,502–68,175 reports were removed.

Table 4 reports the numerical class weights used for the transformer-based classification models. All transformer models used the same weights for the committee-adjudicated dataset because they were trained using the same class distribution. For the full incident reporting system dataset, the weights varied according to the NSE-downsampling ratio selected for each model. Less frequent severity categories received greater weights, allowing them to contribute more strongly to the training objective and supporting more balanced learning across severity categories.

A practical finding from this analysis is that the lower NSE-to-non-NSE ratios were most frequently selected across the evaluated models. The 1:1 ratio was selected for 11 of the 20 models, the 1.5:1 ratio for 8 models, and the 2:1 ratio for one model. Thus, 19 of the 20 models achieved their highest validation Average Precision when the retained NSE population was no more than 1.5 times the combined non-NSE population. For similarly imbalanced incident-reporting datasets, 1:1 and 1.5:1 ratios may therefore provide useful initial configurations. However, variation across models indicates that the final ratio should be selected separately for each architecture and text representation using validation data. For transformer-based models, calculating class weights after downsampling also ensures that the weights reflect the distribution of the data actually used for training.

Table 1. Resampling parameters used for the feature-based classification models across the two study datasets.

| **Parameter** | **Setting** |
| --- | --- |
| **Panel A. Committee-adjudicated dataset** | |
| Resampling method | Synthetic Minority Oversampling Technique (SMOTE) |
| SMOTE implementation | ‘imblearn.over_sampling.SMOTE’ |
| SMOTE sampling_strategy | ‘not majority’ |
| k_neighbors | 5 (default) |
| Resampled classes | LSE, NME, and NSE |
| Synthetic samples generated | LSE (166), NME (785), and NSE (229) |
| Total synthetic samples generated | 1,180 |
| Target sample size | Number of HSE training samples (830) |
| Data partition resampled | Training set only |
| **Panel B. Full incident reporting system dataset** | |
| Resampling method | SMOTE followed by random NSE downsampling without replacement |
| SMOTE implementation | ‘imblearn.over_sampling.SMOTE’ |
| SMOTE sampling_strategy | Class-specific target counts: LSE = 830 and NME = 830 |
| k_neighbors | 5 (default) |
| Resampled classes | LSE and NME |
| Synthetic samples generated | LSE (166) and NME (785) |
| Total synthetic samples generated | 951 |
| Downsampling implementation | ‘sklearn.utils.resample’ |
| Downsampled class | NSE |
| n_samples | Determined by the selected sampling ratio |
| Candidate NSE to non-NSE sampling ratios | [1:1, 1.5:1, 2:1] |
| Downsampling replacement | ‘False’ |
| Classes retained without downsampling | HSE, LSE, and NME |
| Data partition resampled | Training set only |

Table 2. Class-imbalance mitigation parameters used for the transformer-based classification models across the two study datasets.

| **Parameter** | **Setting** |
| --- | --- |
| **Panel A. Committee-adjudicated dataset** | |
| Imbalance-mitigation method | Class-weighted cross-entropy loss |
| Loss implementation | ‘torch.nn.CrossEntropyLoss’ |
| weight | Balanced class weights |
| Weight basis | Severity category frequencies in the training set |
| Data partition used to calculate weights | Training set only |
| **Panel B. Full incident reporting system dataset** | |
| Imbalance-mitigation methods | Random NSE downsampling without replacement followed by class-weighted cross-entropy loss |
| Downsampling implementation | ‘sklearn.utils.resample’ |
| Downsampled class | NSE |
| n_samples | Determined by the selected sampling ratio |
| Candidate NSE to non-NSE sampling ratios | [1:1, 1.5:1, 2:1] |
| Downsampling replacement | ‘False’ |
| Classes retained without downsampling | HSE, LSE, and NME |
| Data partition resampled | Training set only |
| Loss implementation | ‘torch.nn.CrossEntropyLoss’ |
| weight | Balanced class weights |
| Weight basis | Severity category frequencies after NSE downsampling |
| Data partition downsampled and used to calculate weights | Training set only |

Table 3. Selected NSE-to-non-NSE sampling ratios and resulting NSE sample counts by classification model and text representation.

| **Model** | **Selected NSE to non-NSE ratio** | **NSE samples retained** | **NSE samples removed from the training set** |
| --- | --- | --- | --- |
| XGB_TF_IDF | 1:1 | 2,490 | 76,963 |
| XGB_BAG | 1:1 | 2,490 | 76,963 |
| XGB_BIOBERT | 1.5:1 | 3,735 | 75,718 |
| XGB_BERT | 1:1 | 2,490 | 76,963 |
| XGB_ROBERTA | 1.5:1 | 3,735 | 75,718 |
| XGB_GATORTRON | 2:1 | 4,980 | 74,473 |
| LGBM_TF_IDF | 1:1 | 2,490 | 76,963 |
| LGBM_BAG | 1.5:1 | 3,735 | 75,718 |
| LGBM_BIOBERT | 1.5:1 | 3,735 | 75,718 |
| LGBM_BERT | 1:1 | 2,490 | 76,963 |
| LGBM_ROBERTA | 1.5:1 | 3,735 | 75,718 |
| LGBM_GATORTRON | 1.5:1 | 3,735 | 75,718 |
| BERT | 1.5:1 | 2,019 | 67,502 |
| ROBERTA | 1:1 | 1,346 | 68,175 |
| XLM-ROBERTA | 1:1 | 1,346 | 68,175 |
| BIOBERT | 1:1 | 1,346 | 68,175 |
| BIOMEDBERT | 1.5:1 | 2,019 | 67,502 |
| GATORTRON | 1:1 | 1,346 | 68,175 |
| LLAMA3.1 | 1:1 | 1,346 | 68,175 |
| QWEN3 | 1:1 | 1,346 | 68,175 |

**Note:** Feature-based and transformer-based models used different training partitions (80% and 70%, respectively), containing 79,453 and 69,521 NSE samples. For the feature-based models, SMOTE increased the combined number of HSE, LSE, and NME samples from 1,539 to 2,490 before NSE downsampling. The transformer-based models contained 1,346 combined non-NSE samples and did not use SMOTE. Non-NSE samples comprised HSE, LSE, and NME reports. The NSE-to-non-NSE sampling ratio was selected separately for each model–representation combination based on validation Average Precision.

Table 4. Balanced class weights used for transformer-based classification models across the two study datasets.

| **Model** | **Selected NSE-to-non-NSE ratio** | **HSE weight** | **LSE weight** | **NME weight** | **NSE weight** |
| --- | --- | --- | --- | --- | --- |
| **Panel A. Committee-adjudicated dataset** | | | | | |
| BERT | Not applicable | 0.645 | 0.806 | 12.000 | 0.890 |
| ROBERTA | Not applicable | 0.645 | 0.806 | 12.000 | 0.890 |
| XLM-ROBERTA | Not applicable | 0.645 | 0.806 | 12.000 | 0.890 |
| BIOBERT | Not applicable | 0.645 | 0.806 | 12.000 | 0.890 |
| BIOMEDBERT | Not applicable | 0.645 | 0.806 | 12.000 | 0.890 |
| GATORTRON | Not applicable | 0.645 | 0.806 | 12.000 | 0.890 |
| LLAMA3.1 | Not applicable | 0.645 | 0.806 | 12.000 | 0.890 |
| QWEN3 | Not applicable | 0.645 | 0.806 | 12.000 | 0.890 |
| **Panel B. Full incident reporting system dataset** | | | | | |
| BERT | 1.5:1 | 1.159 | 1.448 | 21.571 | 0.417 |
| ROBERTA | 1:1 | 0.927 | 1.158 | 17.256 | 0.500 |
| XLM-ROBERTA | 1:1 | 0.927 | 1.158 | 17.256 | 0.500 |
| BIOBERT | 1:1 | 0.927 | 1.158 | 17.256 | 0.500 |
| BIOMEDBERT | 1.5:1 | 1.159 | 1.448 | 21.571 | 0.417 |
| GATORTRON | 1:1 | 0.927 | 1.158 | 17.256 | 0.500 |
| LLAMA3.1 | 1:1 | 0.927 | 1.158 | 17.256 | 0.500 |
| QWEN3 | 1:1 | 0.927 | 1.158 | 17.256 | 0.500 |

**Note:** Balanced class weights were calculated from the severity-category frequencies in each training dataset and supplied through the weight parameter of torch.nn.CrossEntropyLoss. The committee-adjudicated training partition contained 726 HSE, 581 LSE, 39 NME, and 526 NSE reports. For the full incident reporting system dataset, the training partition contained 726 HSE, 581 LSE, and 39 NME reports, while the NSE class was downsampled to 1,346 or 2,019 reports according to the selected ratio. Class weights for the full incident reporting system dataset were calculated after NSE downsampling and remained fixed throughout model training.
