# Supplementary material for: Machine Learning to Prioritize High-Severity Patient Safety Events for Institutional Investigation: Algorithm Development and Validation Study
Source: JMIR Med Inform. 2026 Sep 29;14:e101393. doi: 10.2196/101393 (PMC13662446; doi:10.2196/101393)
Supplement: Supplementary file 8 [file medinform_v14i1e101393_app6.docx]

## Appendix-6 Mathematical Definitions of Evaluation Metrics.

In this study, we evaluated model performance using established ranking metrics, including average precision (AP), normalized discounted cumulative gain (NDCG) at multiple cutoff thresholds (k = 10, 20, 50, 100, 200), as well as the precision and recall computed at the same cutoffs. The mathematical definitions of these metrics are provided below.

**Average Precision (AP):**

Average Precision is calculated as the mean of the precision values at each rank position where a relevant item (i.e., high-severity PSE) is retrieved. This metric quantifies the model’s ability to place relevant items near the top of the ranked list, thus reflecting the effectiveness of the ranking.

**Formula:**

$$AP=\frac{1}{n}\sum_{i=1}^{N} Precision\left( i \right)*relevance(i)$$

**Definitions:**

- AP: Average Precision.
- n: Total number of relevant items (i.e., high severity PSEs) in the dataset.
- N: Total number of items in the ranked list.
- Precision(i): Precision at rank position i.
- relevance(i): Binary relevance indicator (1 if the item at rank i is relevant, 0 otherwise).

**Normalized Discounted Cumulative Gain (NDCG@k):**

NDCG@k quantifies the quality of a ranked list (length=k) by accounting for both the relevance of items (i.e., severity of PSEs) and their positions in the ranking. Items ranked higher contribute more to the overall score, with their relevance scores discounted logarithmically based on rank. The score is normalized by the Ideal Discounted Cumulative Gain (IDCG@K), which represents the maximum possible DCG@k obtained by ranking all relevant items at the top of the list.

**Formula:**

$$NDCG@k=\frac{DCG@k}{IDCG@k}$$

$$DCG@k=\sum_{i=1}^{k} \frac{2^{r_{i}}-1}{\log_{2} (i+1)}$$

$$IDCG@k=\sum_{i=1}^{k} \frac{2^{{r_{i}}^{*}}-1}{\log_{2} (i+1)}$$

**Definitions:**

- NDCG@k: Normalized Discounted Cumulative Gain at rank k.
- DCG@k: Discounted Cumulative Gain at rank k.
- IDCG@k: Ideal Discounted Cumulative Gain at rank k.
- k: Cutoff threshold (number of top-ranked items considered).
- r_i_ : relevance score at rank i in the predicted order.
- r_i_^*^ : relevance score at rank i in the ideal order (sorted by the highest relevance first)

**Precision@k:**

Precision at rank k quantifies the proportion of relevant items (i.e., high-severity PSEs) among the top k positions in the ranked list. This metric evaluates how accurately the model identifies relevant items within the top k positions.

**Formula:**

$$Precision@k=\frac{1}{k}\sum_{i=1}^{k} relevance(i)$$

**Definitions:**

- Precision@k: Precision at rank k.
- k: Cutoff threshold (number of top-ranked items considered).
- relevance(i): Binary relevance indicator (1 if the item at rank i is relevant, 0 otherwise).

**Recall@k:**

Recall at rank *k* measures the proportion of all relevant items (i.e., high-severity PSEs) that are retrieved within the top *k* positions in the ranked list. The metric reflects the model’s ability to retrieve all relevant items at different cutoff thresholds.

**Formula:**

$$Recall@k=\frac{1}{n}\sum_{i=1}^{k} relevance\left( i \right)$$

**Definitions:**

- Recall@k: Recall at rank k.
- n: Total number of relevant items in the dataset.
- k: Cutoff threshold (number of top-ranked items considered).
- relevance(i): Binary relevance indicator (1 if the item at rank i is relevant, 0 otherwise).
