# Supplementary material for: Machine Learning to Prioritize High-Severity Patient Safety Events for Institutional Investigation: Algorithm Development and Validation Study
Source: JMIR Med Inform. 2026 Sep 29;14:e101393. doi: 10.2196/101393 (PMC13662446; doi:10.2196/101393)
Supplement: Supplementary file 9 [file medinform_v14i1e101393_app7.docx]

## Appendix-7 Evaluation Results of All Developed Ranking and Classification Models.

The tables below summarize the performance of all developed ranking and classification models. Tables 1–4 report the performance and corresponding 95% confidence intervals for the feature-based models, including XGB-CL, XGB-RA, LGBM-CL, and LGBM-RA. Tables 5 and 6 present the performance results for the transformer-based models, with Table 5 showing the classification models and Table 6 showing the ranking models.

Table 1. Performance of the XGB-CL model across multiple evaluation metrics with 95% confidence intervals.

| **Metric** | **XGB_TFIDF** | **XGB_BAG** | **XGB_BIOBERT** | **XGB_BERT** | **XGB_ROBERTA** | **XGB_GATORTRON** |
| --- | --- | --- | --- | --- | --- | --- |
| **Panel A. Committee-adjudicated dataset (SEC-labeled reports)** | | | | | | |
| Average Precision | 0.62±0.07 | 0.68±0.06 | 0.64±0.08 | 0.62±0.08 | 0.65±0.07 | 0.67±0.07 |
| NDCG@10 | 0.98±0.12 | 1.00±0.00 | 0.83±0.17 | 0.76±0.26 | 1.00±0.14 | 0.92±0.16 |
| NDCG@20 | 0.90±0.12 | 1.00±0.01 | 0.89±0.12 | 0.81±0.17 | 0.88±0.13 | 0.95±0.10 |
| NDCG@50 | 0.88±0.08 | 0.94±0.05 | 0.91±0.07 | 0.85±0.11 | 0.88±0.08 | 0.94±0.07 |
| NDCG@100 | 0.84±0.06 | 0.89±0.06 | 0.84±0.07 | 0.84±0.08 | 0.87±0.07 | 0.89±0.06 |
| NDCG@200 | 0.77±0.04 | 0.78±0.05 | 0.77±0.05 | 0.78±0.05 | 0.79±0.05 | 0.81±0.04 |
| Precision@10 | 0.90±0.22 | 1.00±0.00 | 0.70±0.30 | 0.70±0.32 | 1.00±0.20 | 0.90±0.19 |
| Precision@20 | 0.80±0.20 | 1.00±0.04 | 0.85±0.17 | 0.80±0.18 | 0.80±0.20 | 0.95±0.11 |
| Precision@50 | 0.76±0.13 | 0.88±0.10 | 0.86±0.10 | 0.84±0.11 | 0.82±0.10 | 0.90±0.08 |
| Precision@100 | 0.72±0.09 | 0.81±0.11 | 0.74±0.12 | 0.75±0.11 | 0.78±0.10 | 0.76±0.11 |
| Precision@200 | 0.58±0.07 | 0.60±0.07 | 0.59±0.08 | 0.57±0.08 | 0.59±0.08 | 0.60±0.08 |
| Recall@10 | 0.04±0.01 | 0.05±0.00 | 0.03±0.01 | 0.03±0.02 | 0.05±0.01 | 0.04±0.01 |
| Recall@20 | 0.08±0.02 | 0.10±0.01 | 0.08±0.02 | 0.08±0.02 | 0.08±0.02 | 0.09±0.01 |
| Recall@50 | 0.18±0.03 | 0.21±0.03 | 0.21±0.03 | 0.20±0.03 | 0.20±0.03 | 0.22±0.03 |
| Recall@100 | 0.35±0.04 | 0.39±0.05 | 0.36±0.05 | 0.36±0.05 | 0.38±0.05 | 0.37±0.04 |
| Recall@200 | 0.56±0.05 | 0.58±0.06 | 0.57±0.06 | 0.55±0.06 | 0.57±0.06 | 0.57±0.05 |
| **Panel B. Full incident reporting system dataset** | | | | | | |
| Average Precision | 0.43±0.07 | 0.49±0.07 | 0.54±0.06 | 0.55±0.07 | 0.52±0.08 | 0.50±0.07 |
| NDCG@10 | 0.75±0.24 | 1.00±0.10 | 1.00±0.02 | 0.95±0.07 | 0.90±0.07 | 0.66±0.28 |
| NDCG@20 | 0.81±0.16 | 0.92±0.11 | 0.97±0.07 | 0.92±0.07 | 0.90±0.08 | 0.73±0.20 |
| NDCG@50 | 0.81±0.11 | 0.90±0.09 | 0.93±0.06 | 0.91±0.06 | 0.86±0.08 | 0.78±0.13 |
| NDCG@100 | 0.79±0.09 | 0.82±0.07 | 0.81±0.07 | 0.85±0.09 | 0.81±0.08 | 0.76±0.10 |
| NDCG@200 | 0.59±0.08 | 0.65±0.06 | 0.69±0.05 | 0.68±0.06 | 0.71±0.12 | 0.69±0.07 |
| Precision@10 | 0.70±0.28 | 1.00±0.15 | 1.00±0.03 | 0.80±0.23 | 0.80±0.22 | 0.60±0.25 |
| Precision@20 | 0.80±0.16 | 0.90±0.14 | 0.95±0.11 | 0.80±0.16 | 0.80±0.17 | 0.60±0.18 |
| Precision@50 | 0.80±0.11 | 0.86±0.11 | 0.86±0.11 | 0.82±0.11 | 0.76±0.12 | 0.74±0.13 |
| Precision@100 | 0.69±0.10 | 0.67±0.10 | 0.68±0.09 | 0.72±0.13 | 0.68±0.10 | 0.66±0.10 |
| Precision@200 | 0.45±0.08 | 0.48±0.08 | 0.52±0.07 | 0.52±0.07 | 0.55±0.08 | 0.53±0.07 |
| Recall@10 | 0.03±0.01 | 0.05±0.01 | 0.05±0.01 | 0.04±0.01 | 0.04±0.01 | 0.03±0.01 |
| Recall@20 | 0.08±0.02 | 0.09±0.02 | 0.09±0.01 | 0.08±0.02 | 0.08±0.02 | 0.06±0.02 |
| Recall@50 | 0.19±0.03 | 0.21±0.03 | 0.21±0.03 | 0.20±0.04 | 0.18±0.03 | 0.18±0.04 |
| Recall@100 | 0.33±0.05 | 0.32±0.05 | 0.33±0.04 | 0.35±0.05 | 0.33±0.05 | 0.32±0.05 |
| Recall@200 | 0.43±0.06 | 0.46±0.07 | 0.50±0.06 | 0.50±0.05 | 0.52±0.06 | 0.51±0.06 |

Table 2. Performance of the XGB-RA model across multiple evaluation metrics with 95% confidence intervals.

| **Metric** | **XGB_TFIDF** | **XGB_BAG** | **XGB_BIOBERT** | **XGB_BERT** | **XGB_ROBERTA** | **XGB_GATORTRON** |
| --- | --- | --- | --- | --- | --- | --- |
| **Panel A. Committee-adjudicated dataset (SEC-labeled reports)** | | | | | | |
| Average Precision | 0.68±0.06 | 0.65±0.07 | 0.69±0.07 | 0.65±0.07 | 0.69±0.07 | 0.70±0.07 |
| NDCG@10 | 0.93±0.17 | 0.89±0.17 | 0.91±0.17 | 0.72±0.26 | 0.93±0.10 | 0.91±0.18 |
| NDCG@20 | 0.89±0.13 | 0.93±0.12 | 0.90±0.12 | 0.82±0.18 | 0.95±0.08 | 0.93±0.12 |
| NDCG@50 | 0.92±0.08 | 0.89±0.09 | 0.91±0.08 | 0.88±0.10 | 0.90±0.06 | 0.93±0.07 |
| NDCG@100 | 0.85±0.07 | 0.83±0.07 | 0.87±0.06 | 0.84±0.08 | 0.87±0.05 | 0.89±0.07 |
| NDCG@200 | 0.78±0.05 | 0.75±0.05 | 0.81±0.06 | 0.76±0.06 | 0.78±0.04 | 0.80±0.05 |
| Precision@10 | 0.90±0.23 | 0.80±0.21 | 0.80±0.28 | 0.60±0.33 | 0.90±0.20 | 0.90±0.18 |
| Precision@20 | 0.85±0.17 | 0.90±0.13 | 0.85±0.16 | 0.80±0.18 | 0.95±0.12 | 0.90±0.12 |
| Precision@50 | 0.90±0.09 | 0.82±0.11 | 0.88±0.09 | 0.86±0.09 | 0.86±0.10 | 0.88±0.08 |
| Precision@100 | 0.76±0.09 | 0.74±0.09 | 0.81±0.08 | 0.76±0.10 | 0.78±0.08 | 0.82±0.09 |
| Precision@200 | 0.62±0.07 | 0.57±0.08 | 0.66±0.08 | 0.61±0.08 | 0.62±0.08 | 0.63±0.08 |
| Recall@10 | 0.04±0.01 | 0.04±0.01 | 0.04±0.01 | 0.03±0.02 | 0.04±0.01 | 0.04±0.01 |
| Recall@20 | 0.08±0.02 | 0.09±0.02 | 0.08±0.02 | 0.08±0.02 | 0.09±0.01 | 0.09±0.02 |
| Recall@50 | 0.22±0.03 | 0.20±0.03 | 0.21±0.03 | 0.21±0.03 | 0.21±0.03 | 0.21±0.03 |
| Recall@100 | 0.37±0.04 | 0.36±0.05 | 0.39±0.04 | 0.37±0.04 | 0.38±0.04 | 0.40±0.05 |
| Recall@200 | 0.59±0.05 | 0.55±0.06 | 0.64±0.05 | 0.58±0.05 | 0.60±0.06 | 0.60±0.05 |
| **Panel B. Full incident reporting system dataset** | | | | | | |
| Average Precision | 0.47±0.07 | 0.56±0.06 | 0.60±0.08 | 0.57±0.08 | 0.55±0.07 | 0.61±0.07 |
| NDCG@10 | 0.91±0.13 | 1.00±0.07 | 0.86±0.24 | 0.88±0.21 | 0.98±0.05 | 0.93±0.14 |
| NDCG@20 | 0.87±0.11 | 0.92±0.10 | 0.90±0.16 | 0.88±0.14 | 0.95±0.07 | 0.96±0.09 |
| NDCG@50 | 0.87±0.08 | 0.93±0.06 | 0.93±0.09 | 0.91±0.08 | 0.92±0.06 | 0.96±0.06 |
| NDCG@100 | 0.78±0.08 | 0.86±0.06 | 0.90±0.09 | 0.87±0.07 | 0.86±0.07 | 0.92±0.06 |
| NDCG@200 | 0.64±0.06 | 0.68±0.05 | 0.69±0.07 | 0.67±0.07 | 0.68±0.07 | 0.72±0.07 |
| Precision@10 | 0.80±0.23 | 1.00±0.11 | 0.90±0.19 | 0.80±0.25 | 0.90±0.16 | 0.90±0.18 |
| Precision@20 | 0.80±0.17 | 0.85±0.17 | 0.90±0.13 | 0.80±0.18 | 0.90±0.12 | 0.95±0.10 |
| Precision@50 | 0.80±0.10 | 0.88±0.09 | 0.92±0.08 | 0.86±0.10 | 0.88±0.10 | 0.96±0.05 |
| Precision@100 | 0.66±0.12 | 0.80±0.08 | 0.84±0.12 | 0.81±0.09 | 0.76±0.10 | 0.86±0.09 |
| Precision@200 | 0.49±0.08 | 0.54±0.07 | 0.56±0.09 | 0.55±0.09 | 0.52±0.08 | 0.58±0.09 |
| Recall@10 | 0.04±0.01 | 0.05±0.01 | 0.04±0.01 | 0.04±0.01 | 0.04±0.01 | 0.04±0.01 |
| Recall@20 | 0.08±0.02 | 0.08±0.02 | 0.09±0.02 | 0.08±0.02 | 0.09±0.02 | 0.09±0.01 |
| Recall@50 | 0.19±0.04 | 0.21±0.03 | 0.22±0.03 | 0.21±0.04 | 0.21±0.03 | 0.23±0.03 |
| Recall@100 | 0.32±0.05 | 0.39±0.05 | 0.40±0.05 | 0.39±0.05 | 0.37±0.04 | 0.41±0.04 |
| Recall@200 | 0.47±0.06 | 0.52±0.06 | 0.53±0.06 | 0.53±0.07 | 0.50±0.06 | 0.56±0.05 |

Table 3. Performance of the LGBM-CL model across multiple evaluation metrics with 95% confidence intervals.

| **Metric** | **LGBM_TFIDF** | **LGBM_BAG** | **LGBM_BIOBERT** | **LGBM_BERT** | **LGBM_ROBERTA** | **LGBM_ GATORTRON** |
| --- | --- | --- | --- | --- | --- | --- |
| **Panel A. Committee-adjudicated dataset (SEC-labeled reports)** | | | | | | |
| Average Precision | 0.62±0.08 | 0.60±0.07 | 0.63±0.07 | 0.59±0.07 | 0.66±0.06 | 0.62±0.07 |
| NDCG@10 | 0.89±0.15 | 0.98±0.05 | 0.84±0.24 | 0.98±0.05 | 0.97±0.07 | 1.00±0.02 |
| NDCG@20 | 0.92±0.10 | 0.97±0.09 | 0.83±0.17 | 0.92±0.08 | 0.98±0.05 | 0.99±0.06 |
| NDCG@50 | 0.90±0.08 | 0.86±0.08 | 0.89±0.10 | 0.88±0.08 | 0.93±0.05 | 0.88±0.08 |
| NDCG@100 | 0.83±0.07 | 0.84±0.07 | 0.84±0.08 | 0.81±0.06 | 0.83±0.06 | 0.82±0.07 |
| NDCG@200 | 0.73±0.06 | 0.73±0.05 | 0.76±0.06 | 0.74±0.05 | 0.78±0.05 | 0.75±0.06 |
| Precision@10 | 0.80±0.29 | 0.90±0.19 | 0.80±0.25 | 0.90±0.21 | 0.90±0.21 | 1.00±0.07 |
| Precision@20 | 0.85±0.18 | 0.90±0.17 | 0.80±0.16 | 0.75±0.19 | 0.95±0.11 | 0.95±0.15 |
| Precision@50 | 0.86±0.11 | 0.76±0.12 | 0.88±0.08 | 0.72±0.13 | 0.82±0.10 | 0.76±0.13 |
| Precision@100 | 0.74±0.09 | 0.71±0.11 | 0.75±0.10 | 0.66±0.09 | 0.70±0.08 | 0.70±0.09 |
| Precision@200 | 0.56±0.08 | 0.52±0.07 | 0.58±0.07 | 0.54±0.07 | 0.63±0.08 | 0.58±0.08 |
| Recall@10 | 0.04±0.01 | 0.04±0.01 | 0.04±0.01 | 0.04±0.01 | 0.04±0.01 | 0.05±0.01 |
| Recall@20 | 0.08±0.02 | 0.09±0.02 | 0.08±0.02 | 0.07±0.02 | 0.09±0.01 | 0.09±0.01 |
| Recall@50 | 0.21±0.03 | 0.18±0.03 | 0.21±0.03 | 0.17±0.03 | 0.20±0.03 | 0.18±0.03 |
| Recall@100 | 0.36±0.04 | 0.34±0.05 | 0.36±0.05 | 0.32±0.04 | 0.34±0.04 | 0.34±0.04 |
| Recall@200 | 0.54±0.05 | 0.59±0.05 | 0.56±0.05 | 0.52±0.05 | 0.60±0.05 | 0.56±0.06 |
| **Panel B. Full incident reporting system dataset** | | | | | | |
| Average Precision | 0.39±0.07 | 0.44±0.09 | 0.46±0.07 | 0.49±0.06 | 0.45±0.08 | 0.44±0.05 |
| NDCG@10 | 0.97±0.12 | 0.83±0.20 | 0.93±0.14 | 0.91±0.13 | 0.57±0.33 | 0.61±0.23 |
| NDCG@20 | 0.92±0.10 | 0.88±0.14 | 0.94±0.10 | 0.90±0.11 | 0.69±0.22 | 0.50±0.16 |
| NDCG@50 | 0.90±0.08 | 0.90±0.14 | 0.85±0.09 | 0.89±0.07 | 0.77±0.12 | 0.46±0.12 |
| NDCG@100 | 0.76±0.08 | 0.74±0.09 | 0.85±0.09 | 0.78±0.06 | 0.71±0.10 | 0.47±0.08 |
| NDCG@200 | 0.57±0.08 | 0.60±0.07 | 0.74±0.08 | 0.64±0.05 | 0.59±0.08 | 0.43±0.06 |
| Precision@10 | 0.90±0.18 | 0.80±0.27 | 0.90±0.19 | 0.80±0.23 | 0.60±0.32 | 0.70±0.35 |
| Precision@20 | 0.90±0.13 | 0.85±0.17 | 0.90±0.14 | 0.80±0.17 | 0.75±0.18 | 0.55±0.23 |
| Precision@50 | 0.84±0.12 | 0.88±0.11 | 0.76±0.13 | 0.82±0.10 | 0.76±0.13 | 0.52±0.15 |
| Precision@100 | 0.64±0.11 | 0.64±0.11 | 0.63±0.09 | 0.67±0.08 | 0.64±0.12 | 0.56±0.10 |
| Precision@200 | 0.41±0.08 | 0.45±0.09 | 0.44±0.08 | 0.50±0.07 | 0.48±0.09 | 0.50±0.07 |
| Recall@10 | 0.04±0.01 | 0.04±0.01 | 0.04±0.01 | 0.04±0.01 | 0.03±0.02 | 0.02±0.01 |
| Recall@20 | 0.09±0.02 | 0.08±0.02 | 0.09±0.02 | 0.08±0.02 | 0.07±0.02 | 0.03±0.02 |
| Recall@50 | 0.20±0.03 | 0.21±0.03 | 0.18±0.04 | 0.20±0.03 | 0.18±0.03 | 0.07±0.02 |
| Recall@100 | 0.31±0.05 | 0.31±0.05 | 0.30±0.05 | 0.32±0.04 | 0.31±0.05 | 0.15±0.02 |
| Recall@200 | 0.40±0.06 | 0.43±0.07 | 0.42±0.07 | 0.48±0.06 | 0.46±0.07 | 0.27±0.03 |

Table 4. Performance of the LGBM-RA model across multiple evaluation metrics with 95% confidence intervals.

| **Metric** | **LGBM_TFIDF** | **LGBM_BAG** | **LGBM_BIOBERT** | **LGBM_BERT** | **LGBM_ROBERTA** | **LGBM_ GATORTRON** |
| --- | --- | --- | --- | --- | --- | --- |
| **Panel A. Committee-adjudicated dataset (SEC-labeled reports)** | | | | | | |
| Average Precision | 0.64±0.07 | 0.67±0.06 | 0.68±0.07 | 0.65±0.07 | 0.67±0.06 | 0.70±0.05 |
| NDCG@10 | 0.80±0.22 | 1.00±0.01 | 0.71±0.36 | 0.73±0.31 | 1.00±0.02 | 0.98±0.05 |
| NDCG@20 | 0.85±0.17 | 0.99±0.04 | 0.78±0.23 | 0.83±0.21 | 0.98±0.03 | 0.98±0.05 |
| NDCG@50 | 0.87±0.11 | 0.92±0.06 | 0.87±0.13 | 0.83±0.13 | 0.93±0.06 | 0.94±0.06 |
| NDCG@100 | 0.85±0.08 | 0.88±0.05 | 0.84±0.09 | 0.81±0.09 | 0.85±0.05 | 0.90±0.05 |
| NDCG@200 | 0.74±0.05 | 0.76±0.04 | 0.76±0.06 | 0.76±0.06 | 0.79±0.04 | 0.79±0.05 |
| Precision@10 | 0.80±0.22 | 1.00±0.02 | 0.80±0.27 | 0.70±0.28 | 1.00±0.08 | 0.90±0.19 |
| Precision@20 | 0.90±0.15 | 0.95±0.08 | 0.85±0.16 | 0.85±0.18 | 0.90±0.12 | 0.95±0.11 |
| Precision@50 | 0.86±0.10 | 0.88±0.09 | 0.90±0.09 | 0.82±0.11 | 0.84±0.11 | 0.88±0.10 |
| Precision@100 | 0.81±0.10 | 0.81±0.09 | 0.82±0.08 | 0.76±0.09 | 0.74±0.09 | 0.81±0.09 |
| Precision@200 | 0.56±0.08 | 0.56±0.06 | 0.61±0.08 | 0.62±0.08 | 0.60±0.08 | 0.63±0.07 |
| Recall@10 | 0.04±0.01 | 0.05±0.00 | 0.04±0.01 | 0.03±0.01 | 0.05±0.01 | 0.04±0.01 |
| Recall@20 | 0.09±0.02 | 0.09±0.01 | 0.08±0.02 | 0.08±0.02 | 0.09±0.02 | 0.09±0.01 |
| Recall@50 | 0.21±0.03 | 0.21±0.03 | 0.22±0.03 | 0.20±0.03 | 0.20±0.03 | 0.21±0.03 |
| Recall@100 | 0.38±0.04 | 0.39±0.05 | 0.40±0.04 | 0.37±0.04 | 0.36±0.04 | 0.39±0.05 |
| Recall@200 | 0.53±0.05 | 0.54±0.05 | 0.59±0.05 | 0.60±0.06 | 0.58±0.06 | 0.61±0.05 |
| **Panel B. Full incident reporting system dataset** | | | | | | |
| Average Precision | 0.42±0.07 | 0.49±0.07 | 0.55±0.07 | 0.53±0.08 | 0.54±0.07 | 0.60±0.07 |
| NDCG@10 | 1.00±0.00 | 1.00±0.00 | 1.00±0.00 | 0.93±0.07 | 1.00±0.07 | 0.98±0.03 |
| NDCG@20 | 1.00±0.00 | 1.00±0.00 | 1.00±0.01 | 0.95±0.05 | 0.95±0.07 | 0.98±0.03 |
| NDCG@50 | 0.95±0.07 | 0.92±0.06 | 0.88±0.08 | 0.96±0.06 | 0.93±0.05 | 0.95±0.06 |
| NDCG@100 | 0.76±0.10 | 0.79±0.08 | 0.82±0.06 | 0.84±0.07 | 0.84±0.08 | 0.86±0.05 |
| NDCG@200 | 0.52±0.07 | 0.60±0.06 | 0.66±0.05 | 0.65±0.08 | 0.64±0.06 | 0.74±0.06 |
| Precision@10 | 1.00±0.00 | 1.00±0.00 | 1.00±0.00 | 0.70±0.25 | 1.00±0.10 | 1.00±0.14 |
| Precision@20 | 1.00±0.00 | 1.00±0.00 | 1.00±0.01 | 0.85±0.15 | 0.90±0.11 | 0.95±0.10 |
| Precision@50 | 0.92±0.13 | 0.88±0.09 | 0.80±0.12 | 0.92±0.10 | 0.88±0.08 | 0.90±0.11 |
| Precision@100 | 0.63±0.14 | 0.68±0.11 | 0.71±0.09 | 0.76±0.10 | 0.74±0.12 | 0.72±0.08 |
| Precision@200 | 0.38±0.07 | 0.45±0.07 | 0.51±0.07 | 0.50±0.10 | 0.48±0.07 | 0.60±0.08 |
| Recall@10 | 0.05±0.01 | 0.05±0.01 | 0.05±0.01 | 0.03±0.01 | 0.05±0.01 | 0.05±0.01 |
| Recall@20 | 0.10±0.01 | 0.10±0.01 | 0.10±0.01 | 0.08±0.02 | 0.09±0.01 | 0.09±0.01 |
| Recall@50 | 0.22±0.03 | 0.21±0.03 | 0.19±0.03 | 0.22±0.03 | 0.21±0.03 | 0.22±0.03 |
| Recall@100 | 0.30±0.06 | 0.33±0.05 | 0.34±0.05 | 0.37±0.04 | 0.36±0.05 | 0.34±0.05 |
| Recall@200 | 0.36±0.06 | 0.43±0.06 | 0.49±0.05 | 0.48±0.07 | 0.46±0.06 | 0.57±0.06 |

Table 5. Performance of transformer-based classification models (BERT-CL, RoBERTa-CL, XLM-RoBERTa-CL, BIOBERT-CL, BIOMEDBERT-CL, GATORTRON-CL, LLAMA3.1-CL, and QWEN3-CL) across multiple evaluation metrics with 95% confidence intervals.

| **Metric** | **BERT** | **ROBERTA** | **XLM-ROBERTA** | **BIOBERT** | **BIOMEDBERT** | **GATORTRON** | **LLAMA3.1** | **QWEN3** |
| --- | --- | --- | --- | --- | --- | --- | --- | --- |
| **Panel A. Committee-adjudicated dataset (SEC-labeled reports)** | | | | | | | | |
| Average Precision | 0.72±0.06 | 0.73±0.06 | 0.69±0.06 | 0.74±0.06 | 0.71±0.06 | 0.70±0.07 | 0.77±0.05 | 0.75±0.06 |
| NDCG@10 | 1.00±0.04 | 1.00±0.00 | 1.00±0.01 | 1.00±0.00 | 0.96±0.07 | 0.91±0.20 | 1.00±0.02 | 1.00±0.04 |
| NDCG@20 | 0.97±0.07 | 1.00±0.00 | 0.99±0.02 | 1.00±0.00 | 0.98±0.05 | 0.93±0.13 | 1.00±0.04 | 0.98±0.08 |
| NDCG@50 | 0.94±0.06 | 0.96±0.05 | 0.95±0.05 | 0.96±0.04 | 0.94±0.05 | 0.93±0.08 | 0.96±0.05 | 0.95±0.06 |
| NDCG@100 | 0.91±0.05 | 0.91±0.05 | 0.88±0.06 | 0.91±0.05 | 0.91±0.05 | 0.89±0.06 | 0.94±0.03 | 0.92±0.06 |
| NDCG@200 | 0.80±0.04 | 0.80±0.04 | 0.81±0.05 | 0.81±0.04 | 0.80±0.05 | 0.80±0.05 | 0.85±0.05 | 0.82±0.05 |
| Precision@10 | 1.00±0.06 | 1.00±0.00 | 1.00±0.06 | 1.00±0.00 | 0.90±0.19 | 0.90±0.20 | 1.00±0.05 | 1.00±0.08 |
| Precision@20 | 0.95±0.10 | 1.00±0.03 | 0.95±0.10 | 1.00±0.00 | 0.95±0.10 | 0.90±0.13 | 1.00±0.06 | 0.95±0.09 |
| Precision@50 | 0.88±0.09 | 0.92±0.10 | 0.86±0.10 | 0.92±0.07 | 0.90±0.08 | 0.90±0.10 | 0.92±0.09 | 0.90±0.08 |
| Precision@100 | 0.84±0.08 | 0.83±0.07 | 0.80±0.09 | 0.84±0.08 | 0.83±0.09 | 0.82±0.08 | 0.88±0.06 | 0.86±0.09 |
| Precision@200 | 0.65±0.07 | 0.64±0.07 | 0.65±0.08 | 0.66±0.07 | 0.66±0.07 | 0.65±0.09 | 0.71±0.05 | 0.68±0.08 |
| Recall@10 | 0.05±0.01 | 0.05±0.01 | 0.05±0.01 | 0.05±0.01 | 0.04±0.01 | 0.04±0.01 | 0.05±0.01 | 0.05±0.01 |
| Recall@20 | 0.09±0.01 | 0.10±0.01 | 0.09±0.01 | 0.10±0.01 | 0.09±0.01 | 0.09±0.02 | 0.10±0.01 | 0.09±0.02 |
| Recall@50 | 0.21±0.03 | 0.22±0.03 | 0.21±0.03 | 0.22±0.03 | 0.22±0.03 | 0.22±0.03 | 0.22±0.03 | 0.22±0.03 |
| Recall@100 | 0.41±0.04 | 0.40±0.04 | 0.39±0.04 | 0.41±0.04 | 0.40±0.04 | 0.40±0.04 | 0.43±0.04 | 0.42±0.05 |
| Recall@200 | 0.62±0.05 | 0.61±0.05 | 0.62±0.05 | 0.63±0.05 | 0.63±0.05 | 0.62±0.05 | 0.69±0.05 | 0.66±0.05 |
| **Panel B. Full incident reporting system dataset** | | | | | | | | |
| Average Precision | 0.61±0.06 | 0.65±0.07 | 0.52±0.07 | 0.68±0.06 | 0.63±0.07 | 0.62±0.06 | 0.70±0.06 | 0.66±0.05 |
| NDCG@10 | 0.89±0.14 | 0.88±0.13 | 0.94±0.14 | 0.93±0.13 | 0.90±0.16 | 0.92±0.12 | 0.96±0.14 | 0.90±0.10 |
| NDCG@20 | 0.91±0.11 | 0.89±0.10 | 0.79±0.17 | 0.95±0.09 | 0.90±0.13 | 0.90±0.15 | 0.94±0.15 | 0.88±0.12 |
| NDCG@50 | 0.86±0.10 | 0.89±0.07 | 0.76±0.10 | 0.93±0.05 | 0.84±0.09 | 0.86±0.08 | 0.92±0.07 | 0.84±0.06 |
| NDCG@100 | 0.85±0.06 | 0.89±0.06 | 0.76±0.08 | 0.90±0.04 | 0.83±0.07 | 0.88±0.06 | 0.90±0.05 | 0.80±0.05 |
| NDCG@200 | 0.75±0.04 | 0.76±0.05 | 0.68±0.06 | 0.79±0.06 | 0.77±0.05 | 0.75±0.05 | 0.82±0.04 | 0.76±0.05 |
| Precision@10 | 0.80±0.18 | 0.80±0.28 | 0.90±0.20 | 0.90±0.18 | 0.80±0.28 | 0.90±0.14 | 0.90±0.16 | 0.90±0.20 |
| Precision@20 | 0.75±0.18 | 0.85±0.16 | 0.70±0.24 | 0.95±0.10 | 0.75±0.23 | 0.80±0.18 | 0.90±0.14 | 0.86±0.16 |
| Precision@50 | 0.74±0.12 | 0.86±0.10 | 0.58±0.14 | 0.89±0.06 | 0.72±0.13 | 0.78±0.08 | 0.88±0.07 | 0.85±0.10 |
| Precision@100 | 0.71±0.08 | 0.84±0.09 | 0.59±0.10 | 0.84±0.07 | 0.73±0.09 | 0.76±0.08 | 0.86±0.06 | 0.84±0.08 |
| Precision@200 | 0.60±0.05 | 0.59±0.08 | 0.49±0.07 | 0.61±0.09 | 0.63±0.07 | 0.61±0.05 | 0.65±0.06 | 0.64±0.08 |
| Recall@10 | 0.04±0.01 | 0.04±0.01 | 0.04±0.01 | 0.04±0.01 | 0.04±0.01 | 0.04±0.01 | 0.04±0.01 | 0.04±0.01 |
| Recall@20 | 0.07±0.02 | 0.08±0.02 | 0.07±0.02 | 0.09±0.01 | 0.07±0.02 | 0.08±0.02 | 0.09±0.02 | 0.08±0.01 |
| Recall@50 | 0.17±0.04 | 0.21±0.03 | 0.14±0.03 | 0.18±0.03 | 0.17±0.03 | 0.21±0.03 | 0.22±0.03 | 0.21±0.03 |
| Recall@100 | 0.33±0.04 | 0.41±0.04 | 0.29±0.05 | 0.37±0.05 | 0.35±0.05 | 0.40±0.04 | 0.42±0.04 | 0.41±0.05 |
| Recall@200 | 0.60±0.05 | 0.57±0.05 | 0.47±0.05 | 0.61±0.06 | 0.61±0.05 | 0.60±0.05 | 0.63±0.05 | 0.60±0.05 |

Table 6. Performance of transformer-based ranking models (BERT-RA, RoBERTa-RA, XLM-RoBERTa-RA, BIOBERT-RA, BIOMEDBERT-RA, GATORTRON-RA, LLAMA3.1-RA, QWEN3-RA) across multiple evaluation metrics with 95% confidence intervals.

| **Metric** | **BERT** | **ROBERTA** | **XLM-ROBERTA** | **BIOBERT** | **BIOMEDBERT** | **GATORTRON** | **LLAMA3.1** | **QWEN3** |
| --- | --- | --- | --- | --- | --- | --- | --- | --- |
| **Panel A. Committee-adjudicated dataset (SEC-labeled reports)** | | | | | | | | |
| Average Precision | 0.91±0.03 | 0.79±0.05 | 0.67±0.06 | 0.88±0.05 | 0.89±0.04 | 0.81±0.05 | 0.94±0.04 | 0.90±0.02 |
| NDCG@10 | 1.00±0.00 | 1.00±0.01 | 1.00±0.01 | 0.95±0.09 | 1.00±0.00 | 1.00±0.00 | 1.00±0.00 | 1.00±0.02 |
| NDCG@20 | 1.00±0.00 | 0.99±0.02 | 0.99±0.03 | 0.97±0.06 | 0.99±0.03 | 1.00±0.01 | 1.00±0.01 | 0.99±0.03 |
| NDCG@50 | 0.99±0.02 | 0.97±0.03 | 0.92±0.06 | 0.98±0.03 | 0.98±0.02 | 0.98±0.04 | 0.99±0.02 | 0.97±0.03 |
| NDCG@100 | 0.99±0.04 | 0.94±0.04 | 0.88±0.06 | 0.98±0.02 | 0.97±0.02 | 0.93±0.04 | 0.98±0.04 | 0.96±0.05 |
| NDCG@200 | 0.93±0.03 | 0.87±0.03 | 0.81±0.04 | 0.91±0.02 | 0.94±0.03 | 0.88±0.04 | 0.96±0.03 | 0.92±0.04 |
| Precision@10 | 1.00±0.00 | 1.00±0.06 | 1.00±0.04 | 0.90±0.20 | 1.00±0.02 | 1.00±0.00 | 1.00±0.00 | 1.00±0.04 |
| Precision@20 | 1.00±0.00 | 0.95±0.09 | 0.95±0.11 | 0.95±0.10 | 0.95±0.11 | 1.00±0.01 | 1.00±0.02 | 0.95±0.09 |
| Precision@50 | 0.98±0.05 | 0.92±0.07 | 0.82±0.11 | 0.96±0.06 | 0.94±0.07 | 0.98±0.05 | 0.98±0.06 | 0.95±0.08 |
| Precision@100 | 0.97±0.04 | 0.84±0.07 | 0.77±0.09 | 0.95±0.05 | 0.95±0.05 | 0.87±0.07 | 0.96±0.05 | 0.93±0.06 |
| Precision@200 | 0.83±0.07 | 0.74±0.06 | 0.63±0.07 | 0.82±0.06 | 0.85±0.07 | 0.76±0.08 | 0.86±0.06 | 0.82±0.07 |
| Recall@10 | 0.05±0.01 | 0.05±0.01 | 0.05±0.01 | 0.04±0.01 | 0.05±0.00 | 0.05±0.01 | 0.05±0.01 | 0.05±0.01 |
| Recall@20 | 0.10±0.01 | 0.09±0.01 | 0.09±0.01 | 0.09±0.01 | 0.09±0.01 | 0.10±0.01 | 0.10±0.01 | 0.09±0.01 |
| Recall@50 | 0.24±0.03 | 0.22±0.03 | 0.19±0.03 | 0.23±0.03 | 0.23±0.03 | 0.24±0.03 | 0.24±0.03 | 0.23±0.03 |
| Recall@100 | 0.47±0.04 | 0.41±0.04 | 0.37±0.04 | 0.46±0.05 | 0.46±0.05 | 0.42±0.04 | 0.47±0.04 | 0.45±0.04 |
| Recall@200 | 0.80±0.05 | 0.72±0.05 | 0.60±0.06 | 0.81±0.06 | 0.81±0.05 | 0.73±0.05 | 0.83±0.04 | 0.79±0.05 |
| **Panel B. Full incident reporting system dataset** | | | | | | | | |
| Average Precision | 0.87±0.04 | 0.71±0.06 | 0.60±0.07 | 0.74±0.05 | 0.85±0.04 | 0.73±0.06 | 0.89±0.05 | 0.86±0.05 |
| NDCG@10 | 1.00±0.00 | 0.97±0.06 | 0.93±0.14 | 1.00±0.00 | 1.00±0.00 | 1.00±0.03 | 1.00±0.00 | 0.98±0.04 |
| NDCG@20 | 1.00±0.00 | 0.98±0.04 | 0.94±0.10 | 1.00±0.01 | 1.00±0.01 | 0.99±0.03 | 1.00±0.01 | 0.99±0.03 |
| NDCG@50 | 0.99±0.01 | 0.99±0.02 | 0.94±0.06 | 0.99±0.02 | 0.99±0.01 | 0.97±0.04 | 0.99±0.01 | 0.96±0.03 |
| NDCG@100 | 0.96±0.02 | 0.97±0.04 | 0.90±0.05 | 0.98±0.03 | 0.98±0.03 | 0.94±0.05 | 0.98±0.02 | 0.96±0.03 |
| NDCG@200 | 0.94±0.02 | 0.81±0.05 | 0.71±0.06 | 0.83±0.06 | 0.92±0.03 | 0.83±0.05 | 0.95±0.03 | 0.91±0.04 |
| Precision@10 | 1.00±0.00 | 0.90±0.19 | 0.90±0.19 | 1.00±0.00 | 1.00±0.00 | 1.00±0.14 | 1.00±0.00 | 1.00±0.05 |
| Precision@20 | 1.00±0.00 | 0.95±0.10 | 0.90±0.14 | 1.00±0.06 | 1.00±0.04 | 0.95±0.11 | 1.00±0.02 | 0.95±0.10 |
| Precision@50 | 0.97±0.01 | 0.98±0.04 | 0.90±0.09 | 0.96±0.06 | 0.96±0.05 | 0.92±0.08 | 0.98±0.04 | 0.96±0.06 |
| Precision@100 | 0.90±0.06 | 0.92±0.07 | 0.81±0.09 | 0.93±0.07 | 0.95±0.06 | 0.90±0.07 | 0.95±0.05 | 0.91±0.06 |
| Precision@200 | 0.78±0.07 | 0.65±0.08 | 0.55±0.08 | 0.67±0.10 | 0.78±0.08 | 0.67±0.08 | 0.81±0.07 | 0.76±0.07 |
| Recall@10 | 0.05±0.01 | 0.04±0.01 | 0.04±0.01 | 0.05±0.01 | 0.05±0.01 | 0.05±0.01 | 0.05±0.01 | 0.05±0.01 |
| Recall@20 | 0.10±0.01 | 0.09±0.02 | 0.09±0.02 | 0.10±0.01 | 0.10±0.01 | 0.09±0.02 | 0.10±0.01 | 0.09±0.02 |
| Recall@50 | 0.23±0.03 | 0.24±0.03 | 0.22±0.03 | 0.23±0.03 | 0.23±0.03 | 0.22±0.03 | 0.24±0.03 | 0.23±0.03 |
| Recall@100 | 0.43±0.05 | 0.44±0.05 | 0.39±0.04 | 0.45±0.05 | 0.46±0.05 | 0.43±0.05 | 0.46±0.05 | 0.44±0.05 |
| Recall@200 | 0.75±0.06 | 0.62±0.06 | 0.52±0.06 | 0.64±0.06 | 0.74±0.05 | 0.64±0.06 | 0.77±0.05 | 0.73±0.06 |
