# Supplementary material for: Machine Learning to Prioritize High-Severity Patient Safety Events for Institutional Investigation: Algorithm Development and Validation Study
Source: JMIR Med Inform. 2026 Sep 29;14:e101393. doi: 10.2196/101393 (PMC13662446; doi:10.2196/101393)
Supplement: Supplementary file 10 [file medinform_v14i1e101393_app8.docx]

## Appendix-8 Performance of Traditional Classification Baselines Using BAG and TF-IDF Representations.

Table 1 presents the performance of the traditional classification baselines developed using Support Vector Machine (SVM) and Multinomial Naïve Bayes (MNB) with TF-IDF and BAG representations. In the committee-adjudicated dataset, SVM-BAG achieved the highest Average Precision (0.62 ± 0.07), followed closely by SVM-TFIDF (0.61 ± 0.05), and generally demonstrated stronger performance across the evaluated cutoff values. In the full incident reporting dataset, MNB-TFIDF achieved the highest Average Precision (0.49 ± 0.05), followed by SVM-BAG (0.47 ± 0.07). Across both datasets, precision generally decreased and recall increased as cutoff threshold increased, reflecting the expected trade-off between identifying a greater proportion of HSE reports and maintaining precision at broader cutoff values. All traditional baselines were developed and evaluated using the same data partitions and evaluation procedures applied to the other classification models. SVM decision scores and MNB-predicted probabilities for the HSE category were used to order the reports before calculating Average Precision, NDCG@k, Precision@k, and Recall@k.

Table 1. Performance of the traditional classification baselines across multiple evaluation metrics with 95% confidence intervals.

| **Metric** | **SVM_TFIDF** | **SVM_BAG** | **MNB_TFIDF** | **MNB_BAG** |
| --- | --- | --- | --- | --- |
| **Panel A. Committee-adjudicated dataset** | | | | |
| Average Precision | 0.61±0.05 | 0.62±0.07 | 0.59±0.06 | 0.55±0.07 |
| NDCG@10 | 0.93±0.11 | 0.94±0.12 | 0.92±0.15 | 0.90±0.18 |
| NDCG@20 | 0.92±0.10 | 0.93±0.10 | 0.92±0.11 | 0.87±0.14 |
| NDCG@50 | 0.89±0.07 | 0.91±0.08 | 0.86±0.09 | 0.83±0.08 |
| NDCG@100 | 0.80±0.08 | 0.81±0.07 | 0.81±0.07 | 0.77±0.06 |
| NDCG@200 | 0.74±0.05 | 0.72±0.06 | 0.71±0.06 | 0.69±0.05 |
| Precision@10 | 0.90±0.16 | 0.90±0.19 | 0.90±0.21 | 0.70±0.29 |
| Precision@20 | 0.90±0.13 | 0.90±0.14 | 0.90±0.15 | 0.85±0.17 |
| Precision@50 | 0.88±0.10 | 0.89±0.13 | 0.76±0.13 | 0.72±0.12 |
| Precision@100 | 0.67±0.10 | 0.70±0.08 | 0.66±0.11 | 0.59±0.09 |
| Precision@200 | 0.51±0.05 | 0.53±0.06 | 0.50±0.07 | 0.48±0.07 |
| Recall@10 | 0.04±0.01 | 0.04±0.01 | 0.05±0.01 | 0.03±0.01 |
| Recall@20 | 0.09±0.01 | 0.09±0.02 | 0.09±0.02 | 0.08±0.02 |
| Recall@50 | 0.20±0.03 | 0.21±0.03 | 0.18±0.03 | 0.17±0.03 |
| Recall@100 | 0.33±0.04 | 0.35±0.05 | 0.33±0.05 | 0.29±0.04 |
| Recall@200 | 0.50±0.05 | 0.52±0.06 | 0.49±0.06 | 0.48±0.05 |
| **Panel B. Full incident reporting system dataset** | | | | |
| Average Precision | 0.45±0.06 | 0.47±0.07 | 0.49±0.05 | 0.44±0.06 |
| NDCG@10 | 0.71±0.19 | 0.71±0.29 | 0.70±0.30 | 0.72±0.23 |
| NDCG@20 | 0.76±0.15 | 0.64±0.21 | 0.72±0.22 | 0.63±0.17 |
| NDCG@50 | 0.72±0.10 | 0.67±0.13 | 0.68±0.12 | 0.67±0.11 |
| NDCG@100 | 0.68±0.07 | 0.66±0.09 | 0.67±0.09 | 0.70±0.08 |
| NDCG@200 | 0.65±0.06 | 0.65±0.05 | 0.65±0.06 | 0.66±0.05 |
| Precision@10 | 0.60±0.32 | 0.70±0.24 | 0.60±0.03 | 0.60±0.35 |
| Precision@20 | 0.60±0.23 | 0.55±0.23 | 0.60±0.31 | 0.50±0.21 |
| Precision@50 | 0.54±0.14 | 0.50±0.14 | 0.45±0.23 | 0.44±0.13 |
| Precision@100 | 0.49±0.09 | 0.48±0.10 | 0.52±0.14 | 0.42±0.10 |
| Precision@200 | 0.44±0.07 | 0.49±0.07 | 0.50±0.06 | 0.40±0.08 |
| Recall@10 | 0.02±0.02 | 0.03±0.02 | 0.03±0.01 | 0.02±0.02 |
| Recall@20 | 0.05±0.02 | 0.05±0.01 | 0.04±0.02 | 0.04±0.02 |
| Recall@50 | 0.13±0.04 | 0.12±0.03 | 0.13±0.03 | 0.10±0.03 |
| Recall@100 | 0.23±0.04 | 0.24±0.04 | 0.25±0.04 | 0.22±0.05 |
| Recall@200 | 0.42±0.06 | 0.47±0.05 | 0.48±0.06 | 0.41±0.05 |
