# Supplementary material for: Machine Learning to Prioritize High-Severity Patient Safety Events for Institutional Investigation: Algorithm Development and Validation Study
Source: JMIR Med Inform. 2026 Sep 29;14:e101393. doi: 10.2196/101393 (PMC13662446; doi:10.2196/101393)
Supplement: Supplementary file 11 [file medinform_v14i1e101393_app9.docx]

## Appendix-9 Aggregate Error and Model-Disagreement Analysis.

To further examine differences between the best-performing ranking and classification approaches, we conducted an aggregate error and model-disagreement analysis using the committee-adjudicated testing set. The analysis focused on the top 200 reports prioritized by LLAMA3.1-RA and LLAMA3.1-CL. This cutoff represents a fixed review capacity and exhibited a pronounced difference in HSE retrieval between the two models. The 207 HSE reports in the testing set were categorized according to whether they were retrieved by both models, by LLAMA3.1-RA only, by LLAMA3.1-CL only, or by neither model. Table 1 reports these patterns of retrieval agreement and disagreement, including the additional HSE reports retrieved by the ranking model and the cases in which classification succeeded while ranking did not. Table 2 reports the severity-category distribution of the non-HSE reports included within each model’s top 200 positions, distinguishing among LSE, NME, and NSE reports.

Table 1. Aggregate retrieval agreement and disagreement between LLAMA3.1-RA and LLAMA3.1-CL at the top-200 cutoff in the committee-adjudicated testing set.

| **Retrieval Outcome** | **Number of HSE reports** |
| --- | --- |
| Retrieved by both models | 135 |
| Retrieved by LLAMA3.1-RA only | 37 |
| Retrieved by LLAMA3.1-CL only | 7 |
| Retrieved by neither model | 28 |
| Total | 207 |

At the top-200 cutoff, LLAMA3.1-RA retrieved 172 of the 207 HSE reports, whereas LLAMA3.1-CL retrieved 142. Among these reports, 135 were retrieved by both models, 37 were retrieved only by LLAMA3.1-RA, and 7 were retrieved only by LLAMA3.1-CL. The remaining 28 HSE reports were not retrieved by either model. LLAMA3.1-RA therefore recovered 37 of the 65 HSE reports missed by LLAMA3.1-CL (56.9%), whereas LLAMA3.1-CL recovered 7 of the 35 HSE reports missed by LLAMA3.1-RA (20.0%). Overall, the ranking approach produced a net gain of 30 HSE reports within the same 200-report review capacity. These findings demonstrate that the advantage of LLAMA3.1-RA was not limited to assigning higher positions to HSE reports already prioritized by classification. Rather, the ranking model retrieved a substantial number of additional HSE reports that LLAMA3.1-CL did not place within the top 200. This difference is operationally relevant when review capacity is fixed because LLAMA3.1-RA increased HSE retrieval without increasing the number of reports requiring review.

Table 2. Severity-category distribution of non-HSE reports included within the top 200 positions by LLAMA3.1-RA and LLAMA3.1-CL.

| **Model** | **LSE** | **NME** | **NSE** | **Total non-HSE reports included** |
| --- | --- | --- | --- | --- |
| LLAMA3.1-RA | 23 | 0 | 5 | 28 |
| LLAMA3.1-CL | 31 | 1 | 26 | 58 |

Among the 28 non-HSE reports included within the top 200 positions by LLAMA3.1-RA, 23 (82.1%) were LSE reports and 5 (17.9%) were NSE reports; no NME reports were included. In comparison, the 58 non-HSE reports included by LLAMA3.1-CL comprised 31 LSE (53.4%), 1 NME (1.7%), and 26 NSE (44.8%) reports. LLAMA3.1-RA therefore included fewer non-HSE reports overall, and most were from the LSE category, which is closer to HSE within the severity framework. Conversely, LLAMA3.1-CL included substantially more NSE reports within the same top-200 review capacity. Among the non-HSE reports included by LLAMA3.1-RA, reports from the adjacent LSE category occurred substantially more frequently than reports from the lowest-severity NSE category. This pattern indicates that the ranking model produced an ordering that was more closely aligned with the ordinal severity structure across the evaluated review range.
